# Supplementary material for: Collective posterior inference from highly variable empirical replicates
Source: PLoS Comput Biol. 2026 Aug 11;22(8):e1014534. doi: 10.1371/journal.pcbi.1014534 (PMC13475975; doi:10.1371/journal.pcbi.1014534)
Supplement: Supplementary file 1 [file pcbi.1014534.s026.pdf]

Response to Review

Manuscript Number: **PCOMPBIOL-D-26-00248**

Collective Posterior Inference from Highly Variable Empirical Replicates

Dear Editor,

We thank the reviewers for their constructive comments. This revision incorporates their feedback and clarifies several key points. Detailed responses to each comment are provided below in blue.

Sincerely,

Yoav Ram
School of Zoology, Faculty of Life Sciences
Safra Center for Bioinformatics,
Center for Data Science & AI,
Tel Aviv University
yoavram@tauex.tau.ac.il

Reviewer #1

We thank the reviewer for the detailed and thoughtful comments below, which we think significantly help to improve the manuscript.

Overall comments

1. I would like to understand better the dependence on both epsilon and N replicates.

We investigated the dependence on epsilon and N replicates in the following analysis, detailed in the supplementary information (lines 303-314). Briefly, while larger N improves inference quality, epsilon has a larger effect on it.

"**Dependence on ε and N replicates**

To further validate the *Collective Posterior*, we ran the evolutionary benchmark analysis for smaller replicate numbers: 3, 5, or 7 replicates. First, we reran the ε estimation for every single dataset to test the variance across estimations. Estimating ε using the 95th percentile yielded stable results that resemble the single $\varepsilon = e^{-10}$ global estimation (S19 Fig).

Then, we ran the evolutionary benchmark task for all replicate numbers (3, 5, 7, and 10), and for varying epsilon values ($e^{-1000} \leq \varepsilon \leq e^{10}$, S20 Fig). The results show that ε highly affects both the accuracy and the coverage of our method far more than the number of replicates. While increasing the number of replicates slightly improves accuracy and does not affect coverage, ε has an optimal value for accuracy. In addition, while there is no clear optimal ε when we consider both accuracy and coverage, our estimated ε leads to optimal accuracy and a competitive coverage performance (S20 Fig)."

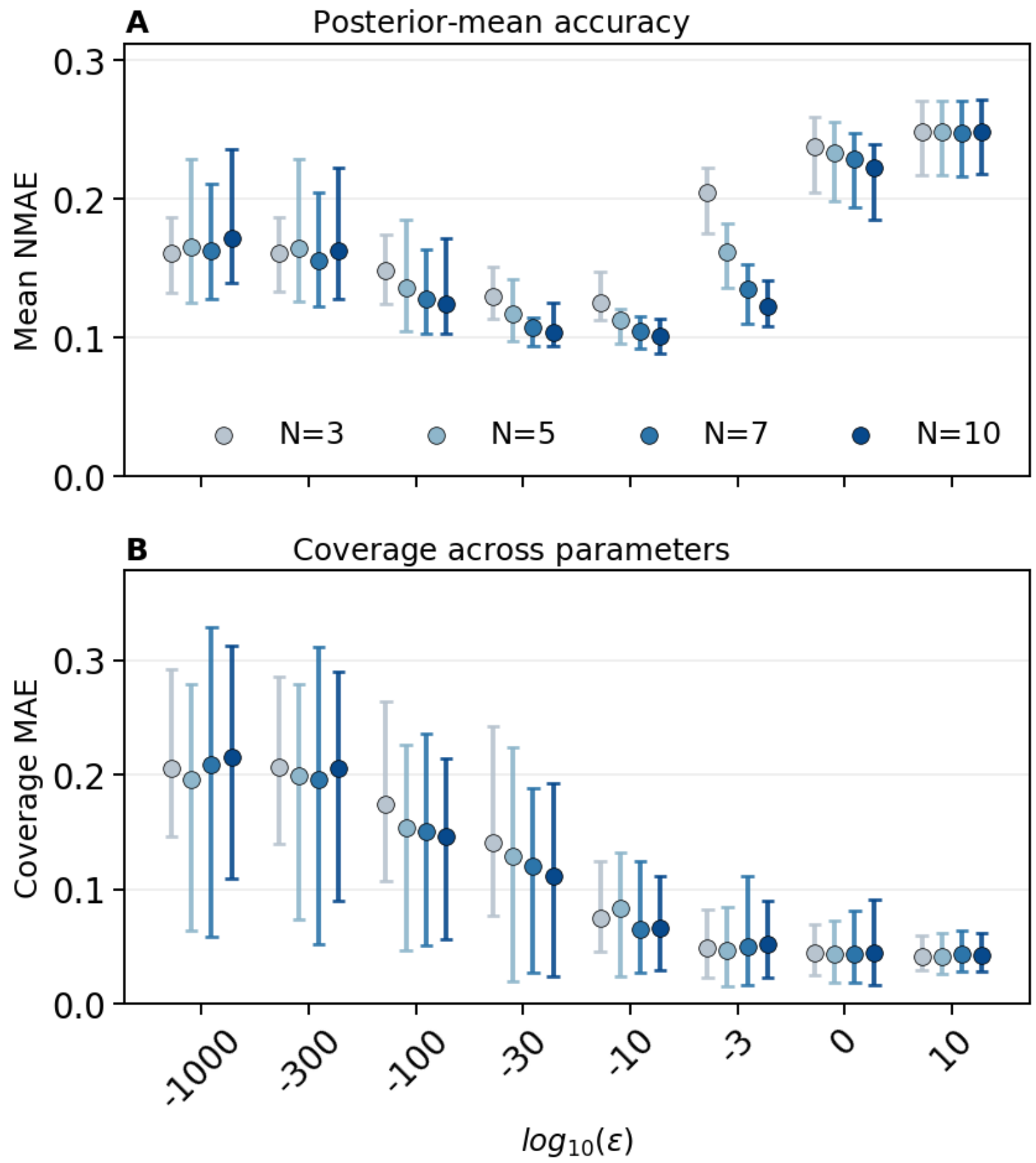

Fig S20. Inference quality is highly dependent on ε. Posterior accuracy (the mean distance between the posterior mean and the true parameter) and mean coverage error as a function of ε and N (number of replicates) for the evolutionary synthetic benchmark. While increasing the number of replicates N can slightly helps both accuracy and coverage, ε has a strong effect on both, requiring a balance between accuracy and coverage optimization. For N=10 we used the original test observations (with 3 outliers out of the 10 replicates). For the other replicate numbers, we sampled randomly (without replacement) from the original 10 replicates, resulting in a varying expected number of outliers (0.9 for 3 replicates, 1.5 for 5 replicates, and 2.1 for 7 replicates). In each panel, the error bars show the between-parameter variance in the respective metric, and the markers show the mean value across parameters.

2. In Fig 5, the method seems to introduce peaks that aren't in the individual posteriors. This should either be explained or investigated further.

The bimodalities are an artifact of the previous sampling approaches we had used (MCMC or SIR with jitter), which were less stable than the adaptive SIR we now use, following a suggestion by one of the reviewers. The revised Fig 5 shows the collective posterior samples obtained using the adaptive SIR sampler. There are no newly introduced peaks.

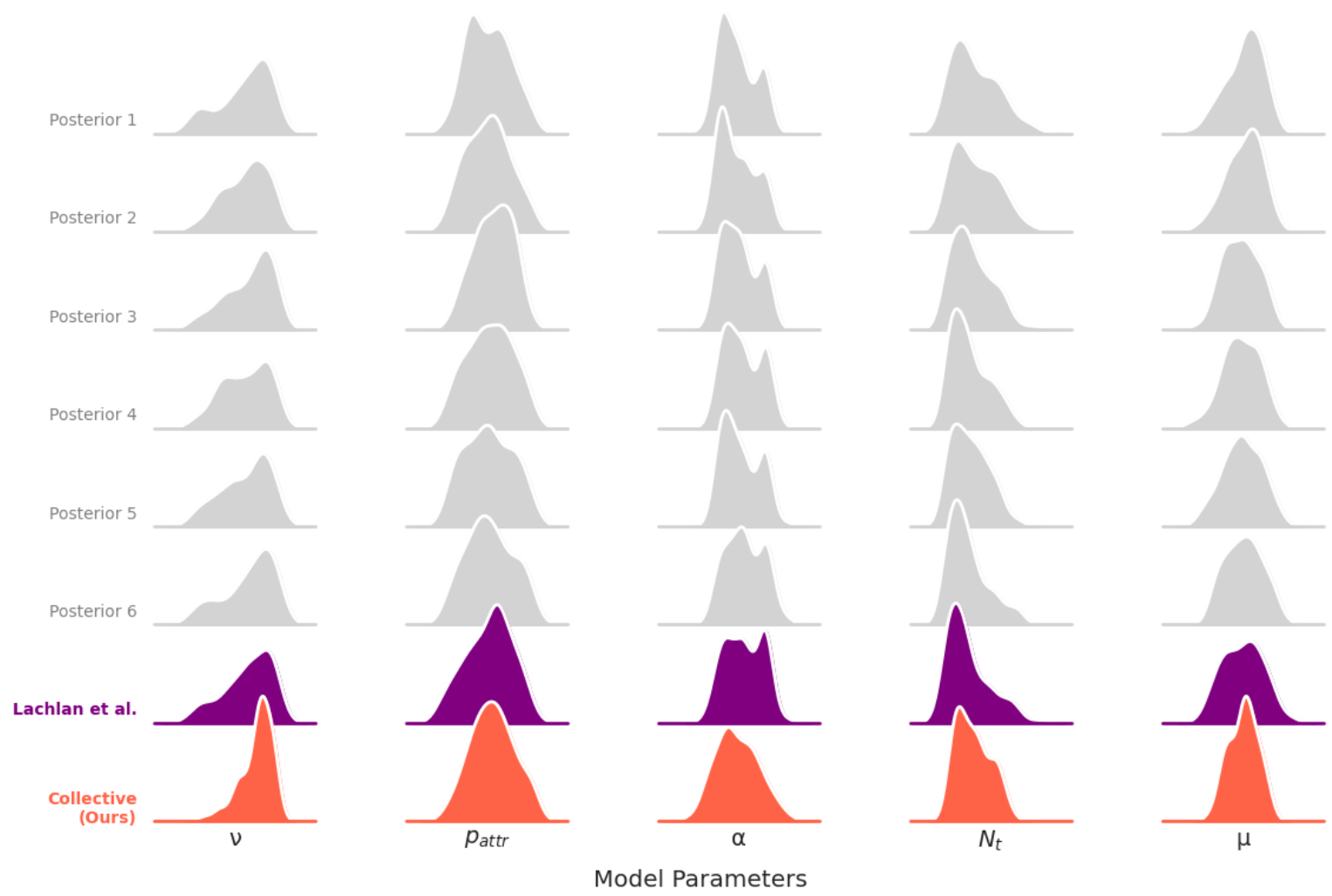

Fig 5. The collective posterior infers cross-population consensus in swamp sparrow cultural evolution parameters. Grey curves show replicate-specific posteriors inferred separately for six populations. The purple curve reproduces the posterior from Lachlan et al. (2018), obtained by conditioning on summary statistics averaged across populations, which yields a comparatively broad posterior. The collective posterior (red) combines replicate-specific posteriors to emphasize parameter regions supported consistently across populations, producing a sharper posterior for parameters with strong cross-population agreement (e.g., ν and μ).

3. While the github repo is very close to being great, it really needs some more work to make installation easier.

We added a requirements file and installation instructions to the repository at https://github.com/yoavram-lab/collective_posterior. We validated the installation on several machines.

I may be misunderstanding, but the adaptive heuristic method seems to just shift the assumed hyperparameter from epsilon to the percentile value.
This isn't necessarily bad, but then it seems like there should be more discussion of the chosen percentiles.

The reviewer correctly notes that the adaptive heuristic replaces the hyperparameter epsilon with a percentile value. We agree that this remains a choice for the user; however, we argue that the percentile is a more robust and intuitive hyperparameter because raw posterior density values can vary by several orders of magnitude depending on the model and parameter dimensionality, and are usually treated as relative. By using a percentile (an inherently relative quantity), we provide a tuning parameter that is stable across different simulators. We have added the following sentence to the Methods section to clarify this – lines 399-401:

> "While this heuristic shifts the hyperparameter selection from a raw density value to a percentile, the latter is more interpretable and stable across different tasks, as it scales naturally with the estimator's dynamic posterior density range."

The interplay of the percentile choice and N replicates seems especially troublesome.
Since the whole point of the method is application to relatively small N, it seems that small changes in epsilon/percentile could make a huge difference in results (by essentially excluding a large fraction of the data).
It seems like choosing the percentile is tantamount to deciding how many outliers to allow, which of course would be bad since we don't know that ahead of time.
Even if I'm mistaken on this point (which would not surprise me!), it would be worth adding explanation of why this isn't the case.

That is a genuine potential concern regarding the interplay between the percentile choice and the number of replicates. We agree that for small N, the robustness of the estimator is critical. However, as shown in the updated Methods and the new Supplementary S19 Fig below, the estimation of ε is based on individual posterior densities and is therefore independent of the total number of replicates N. This means that changing the percentile does not 'exclude' a fixed fraction of the replicates, but rather sets a density "floor" that is stable regardless of sample size N. We have added the following clarifications:

Methods section – lines 409-411:
"Crucially, this estimation of ε remains independent of the number of replicates N, because it is based on the assigned posterior densities given to individual observations."

Results – lines 479-481:
"Importantly, the estimation of ε is independent of the number of replicates in the dataset, since it relies on the densities of posteriors conditioned on individual observations (S19 Fig)."

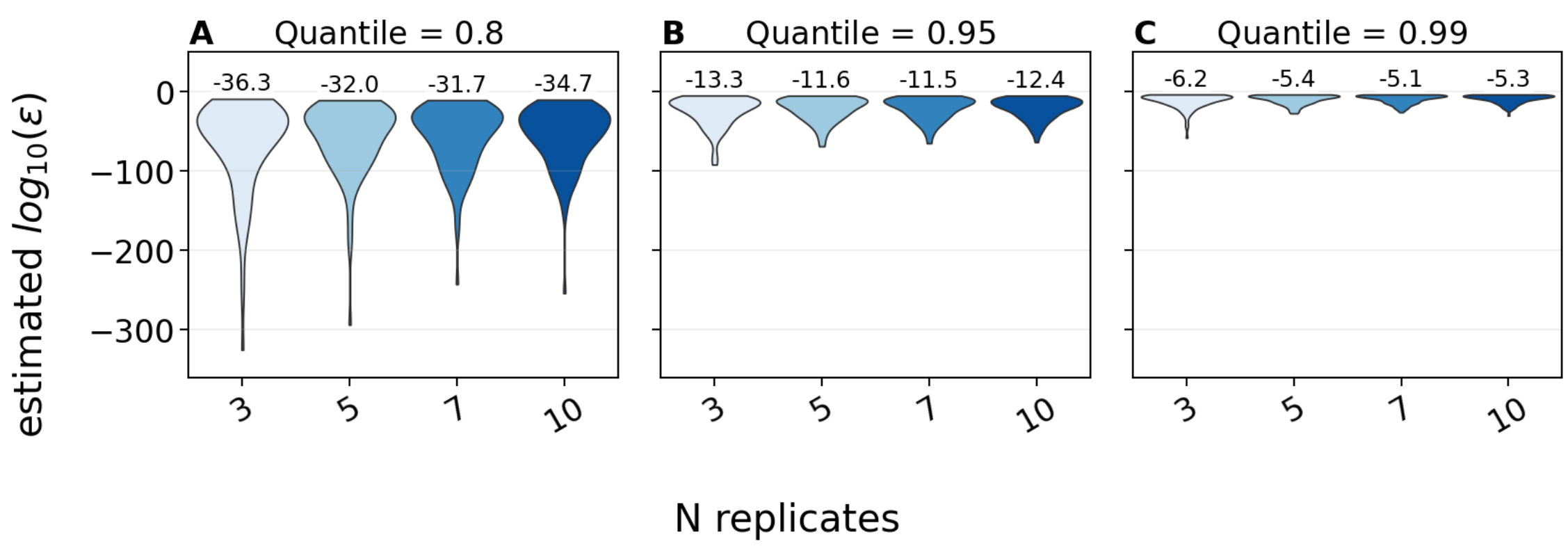

S19 Fig. ε estimation does not depend on the number of replicates N. Distribution of estimated epsilon by quantile choice for the evolutionary benchmark for varying replicate numbers N on 100 test observations. For N=10 we used the original test observations with 3 outliers. For the other replicate numbers, we sampled randomly (without replacement) from the original 10 replicates, resulting in a varying expected number of outliers (0.9 for 3 replicates, 1.5 for 5 replicates, and 2.1 for 7 replicates).The mode of each distribution of estimations is annotated above it.

In practical terms I think it would be worth adding an analysis of how sensitive your results are to epsilon, and maybe also to N replicates.
Fig 4 touches on this, but since it just compares an extremely small epsilon value to a reasonable one, seems to just show that epsilon is important, without much helping to understand the best value.

Both epsilon and N affect inference quality. A larger N mainly improves inference accuracy, while epsilon shows a more interesting relationship with inference accuracy.

We have added an exact analysis of this sensitivity in Fig S20. The analysis clearly shows that while larger N improves inference quality, the effect is negligible compared to epsilon.
Additionally, we added the following texts to the results and SI:

Results – lines 475-483:

"**Dependence on ε and the number of replicates N.** Ideally, we would select an ε that achieves optimal performance. However, identifying an ε that simultaneously balances accuracy and calibration is computationally demanding and can only be rigorously validated on synthetic datasets. We empirically evaluated our percentile-based estimation approach on the evolutionary benchmark. Importantly, the estimation of ε is independent of the number of replicates in the dataset, since it relies on the densities of posteriors conditioned on individual observations (S19 Fig). Moreover, although increasing the number of replicates generally improves inference, the impact of ε on inference quality is both independent of N and substantially greater than the effect of increasing N (S20 Fig)."

SI – lines 303-314:

"**Dependence on ε and N replicates**

To further validate the *Collective Posterior*, we ran the evolutionary benchmark analysis for smaller replicate numbers: 3, 5, or 7 replicates. First, we reran the ε estimation for every single dataset to test the variance across estimations. Estimating ε using the 95th percentile yielded stable results that resemble the single $\varepsilon = e^{-10}$ global estimation (S19 Fig).
Then, we ran the evolutionary benchmark task for all replicate numbers (3, 5, 7, and 10), and for varying epsilon values ($e^{-1000} \leq \varepsilon \leq e^{10}$, S20 Fig). The results show that ε highly affects both the accuracy and the coverage of our method far more than the number of replicates. While increasing the number of replicates slightly improves accuracy and does not affect coverage, ε has an optimal value for accuracy. While there is no clear optimal ε when we consider both accuracy and coverage, our estimated ε leads to optimal accuracy and a competitive coverage performance (S20 Fig)."

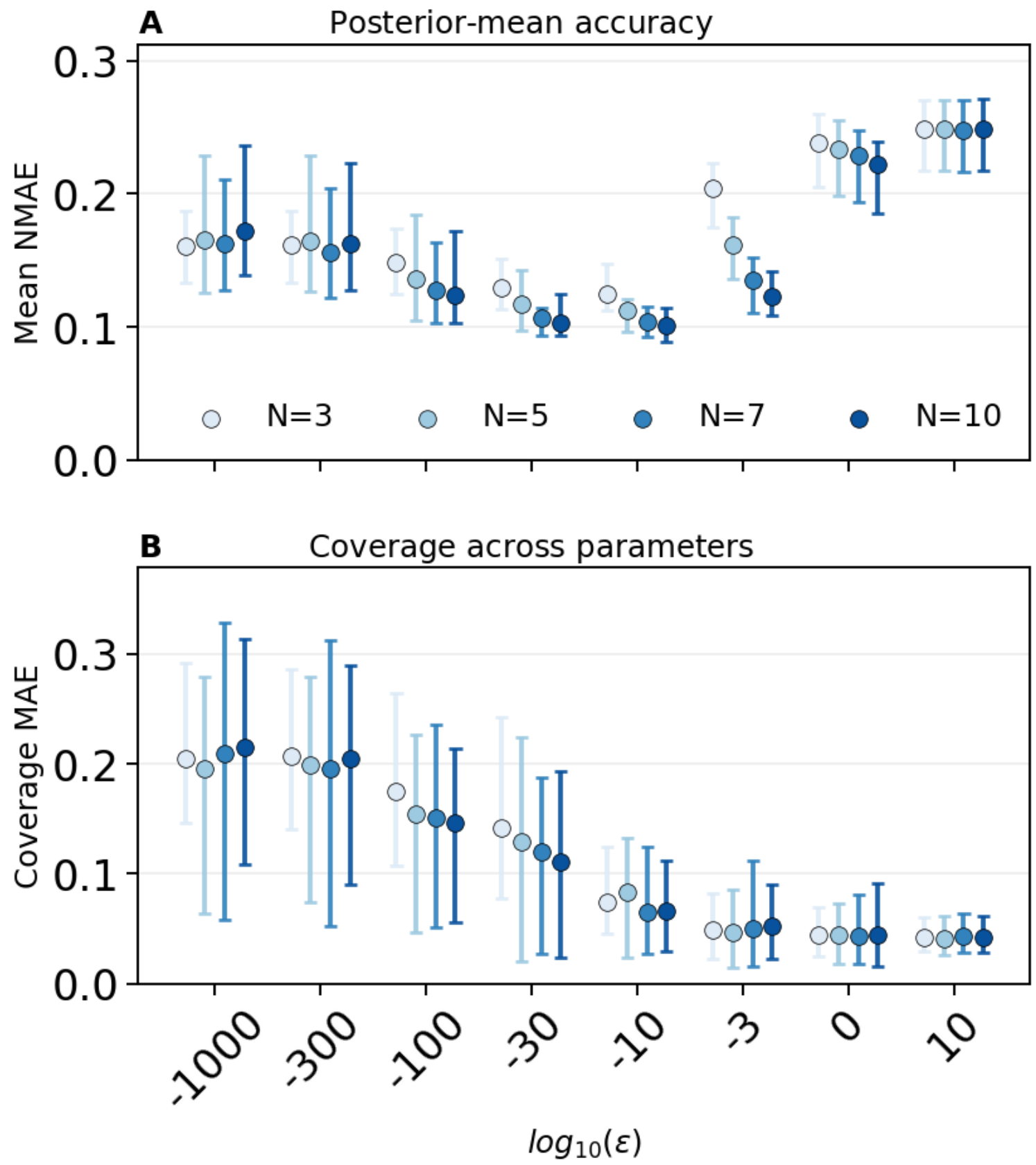

Fig S20. Inference quality is highly dependent on ε. Posterior accuracy (the mean distance between the posterior mean and the true parameter) and mean coverage error as a function of ε and N (number of replicates) for the evolutionary synthetic benchmark. While increasing the number of replicates N can slightly helps both accuracy and coverage, ε has a strong effect on both, requiring a balance between accuracy and coverage optimization. For N=10 we used the original test observations (with 3 outliers out of the 10 replicates). For the other replicate numbers, we sampled randomly (without replacement) from the original 10 replicates, resulting in a varying expected number of outliers (0.9 for 3 replicates, 1.5 for 5 replicates, and 2.1 for 7 replicates). In each panel, the error bars show the between-parameter variance in the respective metric, and the markers show the mean value across parameters.

I also didn't find a discussion of why 10 replicates was chosen.

We have chosen N=10 replicates as it reflects a reasonable biological dataset. We now explain this choice in lines 344-347:

"In previous studies, experimental limitations in biological settings restricted the number of empirical replicates to between three and eight (Lachlan, Ratmann and Nowicki, 2018; Avecilla *et al.*, 2022; Caspi *et al.*, 2023; Chuong *et al.*, 2025; De *et al.*, 2025). However, serial dilution plates are typically designed to facilitate experiments with up to 12 replicates. Therefore, we chose n=10 replicates per observation set as a balanced compromise."

I don't know that every analysis in the paper needs to be redone for multiple N, but there should at least be a discussion of the limits in N of the method's applicability.
It's geared toward "small" N, but surely there's also a lower limit, and this seems important to know from a practical standpoint.

We have analyzed the Collective Posterior's performance for smaller values of N (S20 Fig, see above). We found that while larger N improves inference quality, the performance is relatively similar even for 3 replicates. Therefore, we conclude that our method is useful also useful for smaller datasets

Although they're focused on purposefully splitting up single data samples, it might be worth contextualizing with relation to these papers:
- Minsker et al. (2014, "Robust and Scalable Bayes via a Median of Subset Posteriors"): uses geometric median rather than product, but still with the goal of robustness to outliers https://arxiv.org/abs/1403.2660
- Consensus Monte Carlo approaches, such as: Scott et al. (2016, "Bayes and Big Data: The Consensus Monte Carlo Algorithm")

Thank you for pointing out the connection to divide-and-conquer Bayesian inference methods such as consensus Monte Carlo (Scott et al., 2016) and the median posterior approach of Minsker et al. (2014). We now discuss these methods in the Introduction and clarify both the similarities and differences relative to our approach.

In particular, existing methods primarily focus on scalable inference by partitioning a single large dataset into computational subsets, whereas our setting focuses on combining naturally distinct empirical replicates in simulation-based inference, potentially under model misspecification and replicate heterogeneity. We additionally clarify that our main methodological contribution is the robust density-floor aggregation scheme rather than the posterior factorization identity itself. We have added the following text to the Introduction (lines 131-142):

"Our approach is also conceptually related to divide-and-conquer Bayesian inference methods developed for scalable posterior inference on large datasets, including consensus Monte Carlo (Scott *et al.*, 2022) and median-based posterior aggregation methods (Minsker *et al.*, 2017). These approaches partition a large dataset into subsets, perform inference independently on each subset, and then combine the resulting subset posteriors. However, their primary goal is computational scalability in distributed settings, whereas our setting focuses on aggregating naturally distinct empirical replicates that may exhibit excess variability or model misspecification. In particular, consensus Monte Carlo methods typically combine subset posteriors through averaging or weighted aggregation, while Minsker et al. proposed a geometric median aggregation for robustness to corrupted subsets. Our approach instead uses a robust product-of-experts formulation with a density floor, designed specifically for simulation-based inference from multiple noisy replicates and compatible with amortized neural posterior estimators."

formatting:
Please always include line numbers in drafts.

We added line numbers to the revised version of the manuscript.

Something seems to be wrong with the equation typesetting -- characters are often slightly misaligned.
- e.g. in the Fig 4 caption where a minus sign overlaps an equals sign
- but most or all equations are difficult to read because of the character placement

We separated the minus and equal signs in Fig 4's caption and changed the equation naming. Most of the equations are written as plain text (instead of Word equations) to meet the journal's request.

Equation numbers shouldn't have "eq.", and should be further right. They currently appear to just be part of the equation.

We removed "eq" from the equations and moved the numbering further right.

Fig 2

We re-ran the analysis for Fig 2 with the adaptive SIR sampler, which was implemented following a suggestion by one of the reviewers. Then, we applied all the changes suggested by the reviewer. We believe it helped clarify the analysis.
We reply to each comment below, referring to specific changes in the figure.

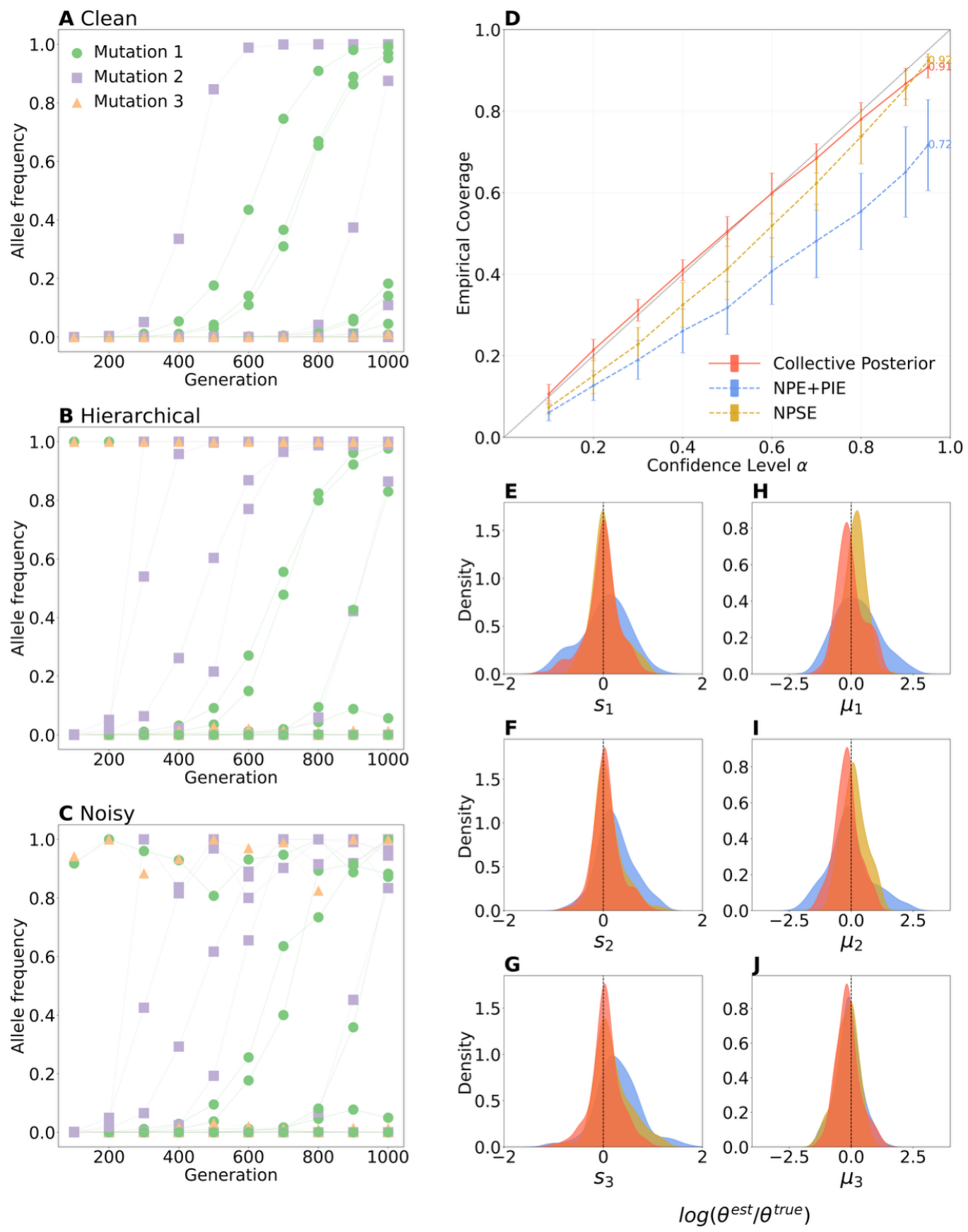

Fig 2. The collective posterior outperforms NPE+PIE and NPSE on noisy evolutionary simulations. (A-C) Synthetic replicate sets. Each test instance includes N=10 replicate time series simulated with shared parameters, θ (A, Clean). To introduce between-replicate heterogeneity, we generate seven replicates using parameters perturbed by moderate Gaussian noise and three replicates using parameters perturbed by larger Gaussian noise (B, Hierarchical). Finally, we add measurement noise to the time series (C, Noisy). **(D) Coverage.** For each nominal confidence level α, we compute the empirical coverage $Pr[\theta_i \in HDR_\alpha]$ of per-parameter highest-density regions (ideal: diagonal). The collective posterior achieved consistently better coverage (closer to the diagonal) than NPE+PIE and NPSE, indicating better calibration. **(E-J) Accuracy.** Distributions of per-parameter estimation errors (defined as the log-ratio of the posterior mean and true value). Across parameters, the collective posterior (red) is more tightly centered around zero, indicating more accurate inference.

- letters indicating panels (a/b/c/d/e) need to be at least bold, if not larger; they're too inconspicuous

The panel's letters are now bold.

panels a, b, c:
- suggest to also put tick labels in a and b

Ticks and labels are present in panels A-C.

- what do red/blue/green indicate? (I don't think it's the same as 2d? But even if it is, 2d doesn't have green)

Colors indicate different genotypes/mutations. It is now described in panel A’s legend.

- it's too hard to distinguish the colors/points -- for instance the green triangles are very hard to see, partly since they're so small that they're hidden by other points

- maybe just larger points and more transparency, but may also need to change colors. Suggest a default contrasting color palette such as 'viridis'

We increased marker size and reduced line width for panels A-C. We also changed the colormap of panels A-C to “Accent”, so it is easier to distinguish between mutations and won’t overlap with the method’s colors.

Fig 2d seems to over-cover, which is better than under-covering, but still not optimal

The underconfidence of the collective posterior, indicated by the above-diagonal values, is usually considered better than the overconfidence of NPSE and NPE+PIE. However, using the adaptive SIR sampler, we do not see this underconfidence. The collective posterior is almost ideally calibrated.

- is this related to epsilon choice?

The coverage of our method depends on epsilon choice. We have carefully studied it as a part of the revision, see S20 Fig above.

- maybe epsilon choice should explicitly target correct coverage?
- please comment on and explain this

Ideally, we would choose an epsilon that yields top performance. However, this is unfeasable in practice due to the time it takes to find this optimal value, since it requires testing the method on many synthetic observations. Our

heuristic does lead to a near-top performance on this benchmark (S20 Fig), and a state-of-the-art performance on the empirical data from Chuong et al., 2025 (Fig 3). We also discuss it in the results section (lines 475-483):

"**Dependence on ε and the number of replicates N.** Ideally, we would select an ε that achieves optimal performance. However, identifying an ε that simultaneously balances accuracy and calibration is computationally demanding and can only be rigorously validated on synthetic datasets. We empirically evaluated our percentile-based estimation approach on the evolutionary benchmark. Importantly, the estimation of ε is independent of the number of replicates in the dataset, since it relies on the densities of posteriors conditioned on individual observations (S19 Fig). Moreover, although increasing the number of replicates generally improves inference, the impact of ε on inference quality is both independent of N and substantially greater than the effect of increasing N (S20 Fig)."

This sentence confuses me, I think there's some misplaced words:
"we perturb θ using for 7 replicates with moderate Gaussian noise and for 3 replicates with larger perturbations (B, Hierarchical)"

Thank you for finding this typo. We have updated Fig 3's caption (addition is underlined, see the revised Fig 2 above).

If the colors do mean the same thing in all panels, the legend needs to include all colors (green?) and be outside a panel/in first panel
- if so, need to use the exact same colors in all panels (the red/blue in a-c are different to those in the other panels)
- if not, use more different colors for different panels

We switched the colors for the example observations to distinguish them from the methods' colors. In addition, we added a legend in panel A.

Fig 3
CNV should be defined in caption

Copy number variant (CNV) is now defined in the caption:
"**Fig 3. The collective posterior accurately captures the between-replicate variation in empirical evolutionary data. (A-D)** For each of the four datasets from Chuong et al. (2025), which follow the evolution of copy number variants (CNV) in yeast populations, we simulate 50 time series using parameter sets sampled from the collective posterior with NPE (red), NPE+PIE (blue), and NPSE (yellow), and compare them to the empirical replicates (grey). The collective posterior more closely captures the observed between-replicate variability. Here, ε is estimated separately for each replicate set. **(E)** Predictive coverage, i.e., the deviation from ideal prediction-to-data calibration. **(F)** Inference stability, i.e., the overlap between the estimated posteriors (from empirical vs. synthetic data)."

I think I'm supposed to be just visually comparing the spread of four different groups of lines here?
I can kind of tell that yellow and red are bundled more tightly than grey, but:
- the plots are too busy -- I can't see much because so many lines are on top of each other
- the hardest to see is empirical (grey)
- Suggest at least changing the empirical color to be easier to see, and/or adding transparency to all of them
- It may be better to add a lightly shaded band for each method (leave empirical as single lines), rather than plotting all curves for every method
- I don't think just looking at visual scatter is sufficient to make a claim as strong as "more closely captures the observed between-replicate variability".
- To justify this I think you'd need to quantify the variance of each method here.

We implemented the reviewer's suggestions and quantified the prediction quality using predictive coverage and an overlap index. We also slightly changed the proportions of the figure, and we think it improves visual clarity. We explained the metrics in the methods and the results, and added two panels to Fig 3.

Methods – lines 363-386:

"**Evaluation metrics with empirical data.** To assess whether each method produced calibrated predictions for the empirical trajectories, we computed its *posterior predictive coverage* (Leininger and Gelfand, 2017). For each dataset and inference method, we drew parameter samples from the inferred posterior and simulated frequency trajectories with these parameter samples. At each nominal coverage level $\alpha \in \{0.1, 0.2, \ldots, 0.9, 0.95\}$, we formed central posterior predictive intervals by taking the $(1-\alpha)/2$ and $(1+\alpha)/2$ quantiles of the simulated trajectories at each generation. We then computed the fraction of empirical observations that fell within these intervals. A calibrated posterior predictive distribution should have empirical coverage close to the nominal level. We summarized calibration error as the mean absolute deviation between empirical and nominal coverage across all nominal levels. We plotted 1-MAE as a coverage score, where larger values indicate better predictive calibration. To quantify whether posterior estimates were stable, we used an *inference cycle* (Cook, Gelman and Rubin, 2006; Säilynoja *et al.*, 2026): first, we inferred the posterior distribution conditioned on the empirical data (5-8 replicates). Then, we used 10 samples from the posterior distribution to simulate a synthetic dataset of 10 replicates. We inferred a posterior distribution conditioned on the synthetic dataset and compared it to the original posterior (conditioned on the empirical data). For each parameter, we computed the *overlapping coefficient* (Weitzman's Δ, or OVL) (Pastore and Calcagnì, 2019) between the marginal posterior distributions,
$OVL(p, q) = \int \min[p(\theta), q(\theta)]\, d\theta$, where p is the original posterior marginal and q is the inference-cycle posterior marginal. The OVL ranges from 0 to 1, with 0 indicating no overlap and 1 indicating identical marginal distributions. In practice, we estimated the OVL from posterior samples by binning both sample sets on the same parameter range, normalizing the bin counts to probability masses, and summing the minimum probability mass in each bin. We report the mean OVL across parameters as a metric for inference-cycle stability, where larger values indicate greater agreement between the original and cycle posteriors."

Results – lines 437-443:

"Using NPE as the replicate-level posterior estimator, the collective posterior produced accurate posterior predictive timeseries across datasets (Fig 3). In addition, it achieved the best predictive coverage score on all four datasets (Fig 3C) and consistently produced stable posterior distributions (Fig 3D). NPSE performs similarly well (and maybe better) in terms of stability, however was significantly less calibrated to the empirical datasets. NPE+PIE yielded inaccurate posterior predictive timeseries for two of the four datasets (Fig 3AB)."

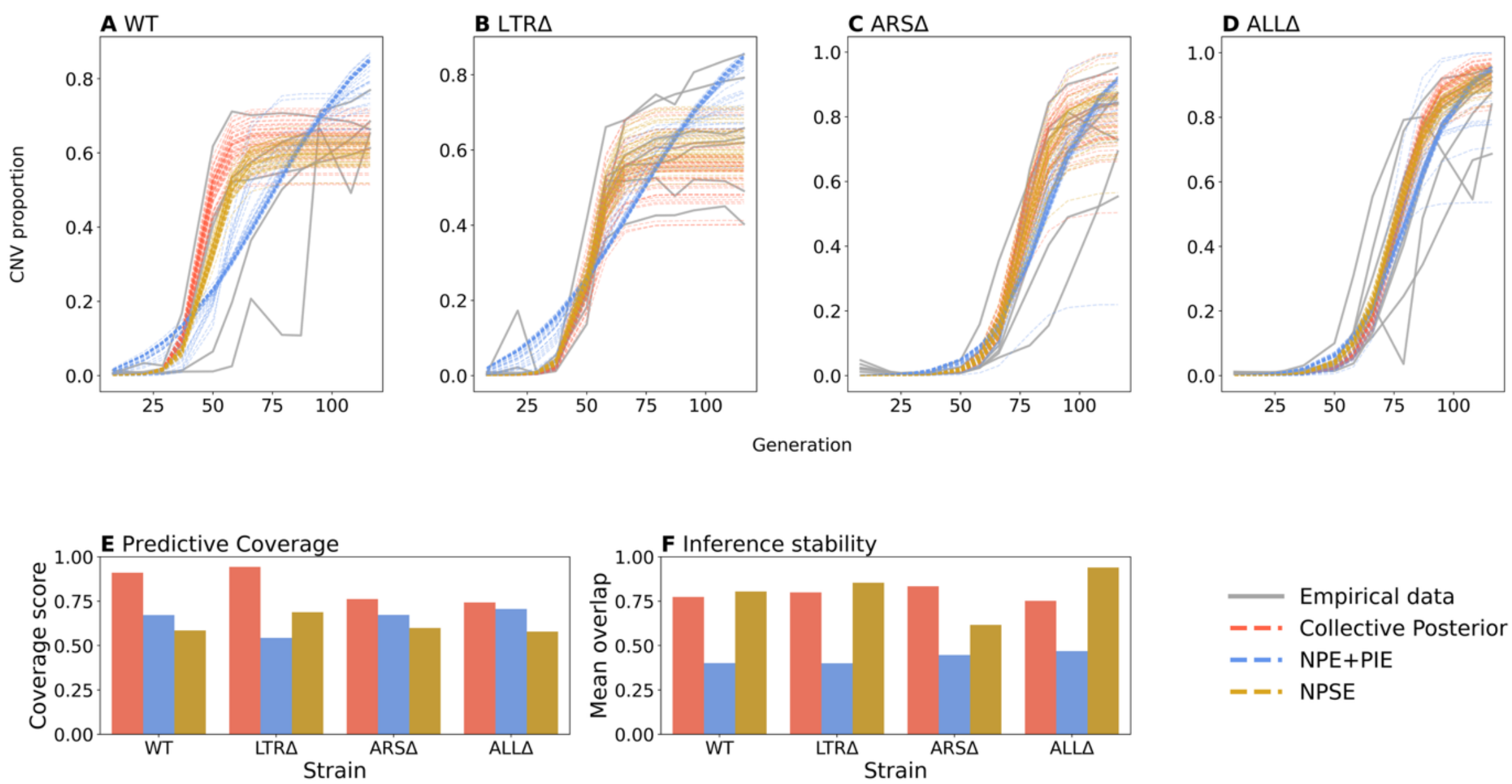

Fig 3. The collective posterior accurately captures the between-replicate variation in empirical evolutionary data. (A-D) For each of the four datasets from Chuong et al. (2025), which follow the evolution of copy number variants (CNV) in yeast populations, we simulate 50 time series using parameter sets sampled from the collective posterior with NPE (red), NPE+PIE (blue), and NPSE (yellow), and compare them to the empirical replicates (grey). The collective posterior more closely captures the observed between-replicate variability. Here, ε is estimated separately for each replicate set. **(E)** Predictive coverage, i.e., the deviation from ideal prediction-to-data calibration. **(F)** Inference stability, i.e., the overlap between the estimated posteriors (from empirical vs. synthetic data).

Fig 4

In its current form, this figure seems more suited to earlier in the text, perhaps the introduction, since it's just establishing that espilon helps minimize the effects of outliers (i.e. providing some intuition for the equations).

I think it would be better, however, to have a comprehensive test of the effects of different epsilon choices.

We reviewed Fig 4's placement in the text, and concluded that it's main purpose is to demonstrate the application of epsilon. Therefore, we decided to keep it as part of the Results.

Following the reviewer's suggestion, we revised Fig 1 to better describe the collective inference framework use of epsilon to handle extreme replicates..

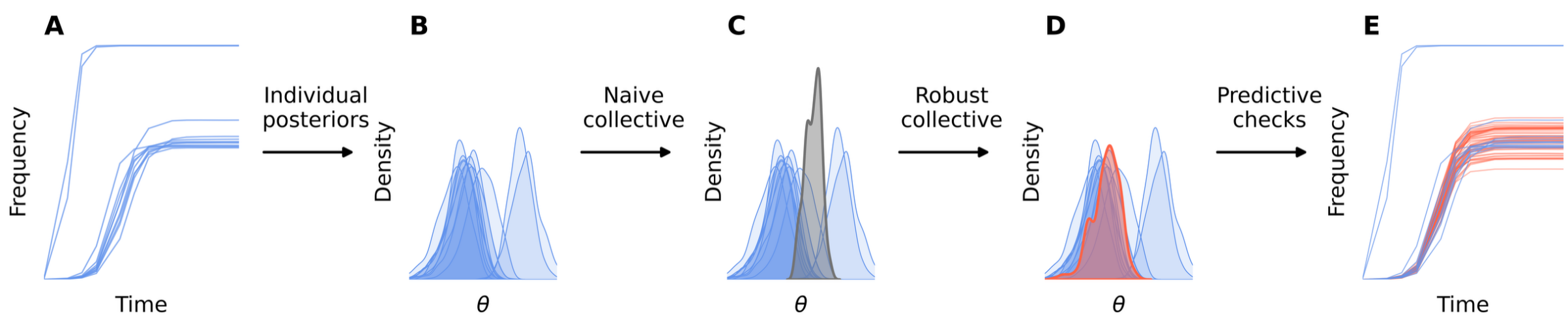

Fig 1. Collective posterior inference. (A) First, we are given a set of independent empirical replicates (e.g., time series; blue), possibly including outliers. **(B)** A density estimator infers replicate-specific posterior distributions (blue). **(C)** The naïve solution would combine the individual posterior distributions to form a collective posterior conditioned on the full replicate set (grey). **(D)** Instead, we use the ε-flooring to form a robust collective posterior distribution (red). **(E)** Finally, posterior predictive checks assess whether collective-posterior predictions (red) reproduce the empirical distribution of time series across replicates (blue), including between-replicate variability.

Fig 5

Three extra distributions (nu, N_t, and mu) are bimodal in the collective posterior, but not in either the individual posteriors or the purple method. Is this worrisome? Is it expected?

Some of the extra peaks really don't look like they correspond to any feature in the replicate-specific data.

Why is it necessarily good that your method gives narrower posteriors?

The text quotes a formula for how much narrower, but this should be compared to the widths in the Figure, since I would guess based on the over-coverage above that they might be too narrow.

The observed bimodalities were an artefact of the sampling method for the collective posterior. For assuring stability, we switched to the new adaptive SIR sampler (see above). This resulted in a unimodal posterior, as displayed in the revised Fig 5.

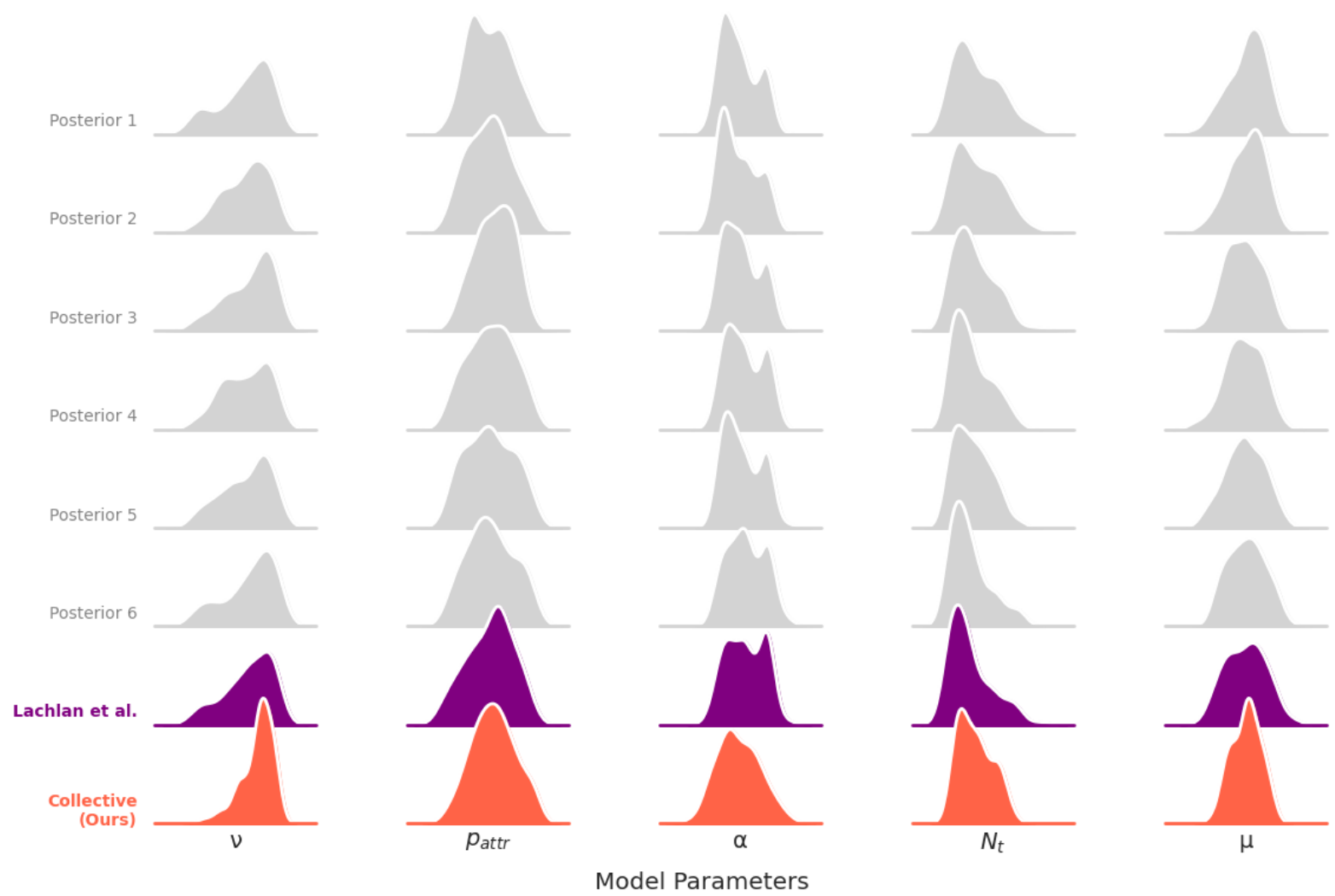

Fig 5. The collective posterior infers cross-population consensus in swamp sparrow cultural evolution parameters. Grey curves show replicate-specific posteriors inferred separately for six populations. The purple curve reproduces the posterior from Lachlan et al. (2018), obtained by conditioning on summary statistics averaged across populations, which yields a comparatively broad posterior. The collective posterior (red) combines replicate-specific posteriors to emphasize parameter regions supported consistently across populations, producing a sharper posterior for parameters with strong cross-population agreement (e.g., ν and μ).

p20

This seems like the first you're mentioning this alternative concatenation approach -- how does it relate to all the other methods you mention in the introduction?

The method we refer to is a trivial alternative from the family of NPE+PIE. However, instead of a permutation invariant embedding, it uses no embedding – we feed the N replicates as a single "flat" vector to the density estimator. Since this method is not worth exploring in the future, we moved this text to the introduction, lines 87-93:

"A direct strategy is to explicitly model the full hierarchical data-generating process and concatenate the replicates into a single "super observation" vector, followed by inference on the global parameters from this high-dimensional input. However, posterior estimation generally becomes harder as the dimensionality of the data increases (curse of dimensionality), both for neural and non-neural density estimators (Fearnhead and Prangle, 2012; Greenberg, Nonnenmacher and Macke, 2019; Lueckmann *et al.*, 2021). Consistent with this, Avecilla et al. (Avecilla *et al.*, 2022) found that concatenating replicates reduced inference accuracy when estimating selection coefficients from evolutionary experiments."

reproducibility

While I very much appreciate the authors' effort to get everything up on github, it would really benefit from a little more work to make it easier to install.

I got the code to run, but it required a lot of trial and error.

The readme says sbibm isn't required, but simulators.py imports sbibim at top level, so importing any simulator crashes without it, so I had to install it.

Following the version specifications in the readme doesn't seem to work

- sbibm requires sb < 0.22.0, but the pretrained posterior pkl files were saved with sb >= 0.23, so the pickle load calls crash
- the workaround seems to be to force-install sbi==0.23.1 after sbibm, breaking sbibm's version constraint
- the readme's install order does work (install sbibm first, then override with sbi==0.23.1), but pip warns about the incompatibility, so it would be nice to mention this

It would be great to have a requirements.txt, setup.py, or pyproject.toml, so we don't have to guess on dependencies, ideally incorporating into a pypi package.

We investigated the installation issue and validated it on several independent machines. The README.md and requirements.txt files were updated to correctly provide installation instructions.

Reviewer #2

Firstly, I apologize for the delay in returning my review. Let me say openly that I do not use LLMs, at all, in peer review. This means that it takes me much longer than many others, and there are likely elements that I may miss. I am enough of a purist that I believe that peer review should be done by humans, now at least (until the scientific community converges on a formal policy).

We appreciate you in taking the time and effort to review our manuscript!

I enjoyed this manuscript overall and believe that an iteration of it should be fit for publication in the near future. That is, I feel like the question that it addresses is relevant; I feel like it handles most of the technical details at least reasonably well; I enjoyed the multiple use cases. But there are several issues that need to be addressed before it is suitable for publication, in my view. I'll divide my comments into "Major" and "Minor" comments.

Thank you for this favorable review and for the following comments.

MAJOR COMMENTS

This manuscript addresses an important and practical problem in simulation-based inference: how to combine information from multiple empirical replicates when likelihoods are intractable and replicate variability exceeds what the generative model predicts. Put bluntly, this is one of the most important problems in all of empirical biology and is related to the highly popularized "replication crisis." While this study may not solve this issue, it does offer some very good ideas. The paper proposes a simple and general approach that aggregates replicate-level posterior distributions into a single collective posterior using a product-of-experts formulation, with an additional mechanism designed to reduce sensitivity to outliers.

I enjoyed this paper and think the core idea is genuinely useful. Aggregating replicate-level posteriors via a product-of-experts is simple, broadly applicable, and the biological use cases make a compelling story. I'd like to see it published, but there are a few things that need addressing first.

On the methodology. The derivation of the collective posterior rests on assumptions (conditional independence of replicates, a shared prior) that are never explicitly stated. Please spell these out.

We have revised the methods section to explicitly state the assumptions and limitations of our method. While the original text mentioned the conditional independence assumptions, we further clarified that the prior is shared (lines 206-211):

"**Standard collective posterior distribution.** Assuming that multiple replicates $\{x_i\}^{n}_{i=1}$ are independent conditioned on the model parameters, θ, that is, $p(x_i,x_j|\theta) = p(x_i|\theta)p(x_j|\theta)$, we can compute the *collective* posterior distribution of model parameters conditioned on all replicates, $p(\theta|x_1,\ldots,x_n)$, from individual posterior distributions,

p(θ|x_i), and the shared prior distribution, p(θ), by applying Bayes' theorem twice (a full derivation is available in the SI),

$$p(\theta|x_1,\dots,x_n) = \frac{p(\theta)^{1-n}\Pi_i[p(\theta|x_i)]}{\int p(\xi)^{1-n}\Pi_i[p(\xi|x_i)]\,d\xi} \quad (1)$$ "

Similarly, the justification for temperature scaling leans on a variance argument that only holds cleanly for Gaussians. A bit more support here, even empirical, would help.

Following a reviewer comment, we revised the sampling method and now use an adaptive SIR sampler (lines 236-248). This sampler automatically fits a temperature to the data to ensure a minimal desired effective sample size.

"**Sampling Importance Resampling (SIR).** To efficiently approximate the collective posterior without relying on sequential chains (i.e., MCMC), we used an SIR scheme (Smith and Gelfand, 1992). We used the prior distribution p(θ) as the proposal distribution to ensure robust coverage of the parameter space while not relying on the sampling efficiency of the individual posterior distribution. Importance weights were calculated as the ratio of the unnormalized robust collective posterior density to the prior density. To counteract the artificial variance reduction inherent in the product-of-experts aggregation, where multiplying independent densities shrinks the variance, we used an adaptive temperature scaling to the importance weights. This temperature was determined via binary search to maintain a target *effective sample size* (ESS), preventing weight collapse as the number of replicates increased. Then, at resampling, we applied Gaussian jittering to the results. This vectorized approach enabled rapid, high-throughput posterior approximation. While practical and accurate, our SIR scheme is designed for high-performance approximation rather than the asymptotic sampling guarantees of MCMC."

On the benchmarking. For a methods paper, the comparison feels thin. NPE+PIE is the primary competitor, and NPSE is dismissed fairly quickly. It would strengthen the paper to engage more seriously with the broader landscape of competing approaches.

While NPE+PIE is the primary competitor, we agree that NPSE is an alternative worth exploring. We moved the comparison to NPSE to the main text. We now include it in Fig 2. The collective posterior is more calibrated than NPSE while achieving a similar accuracy in parameter recovery.

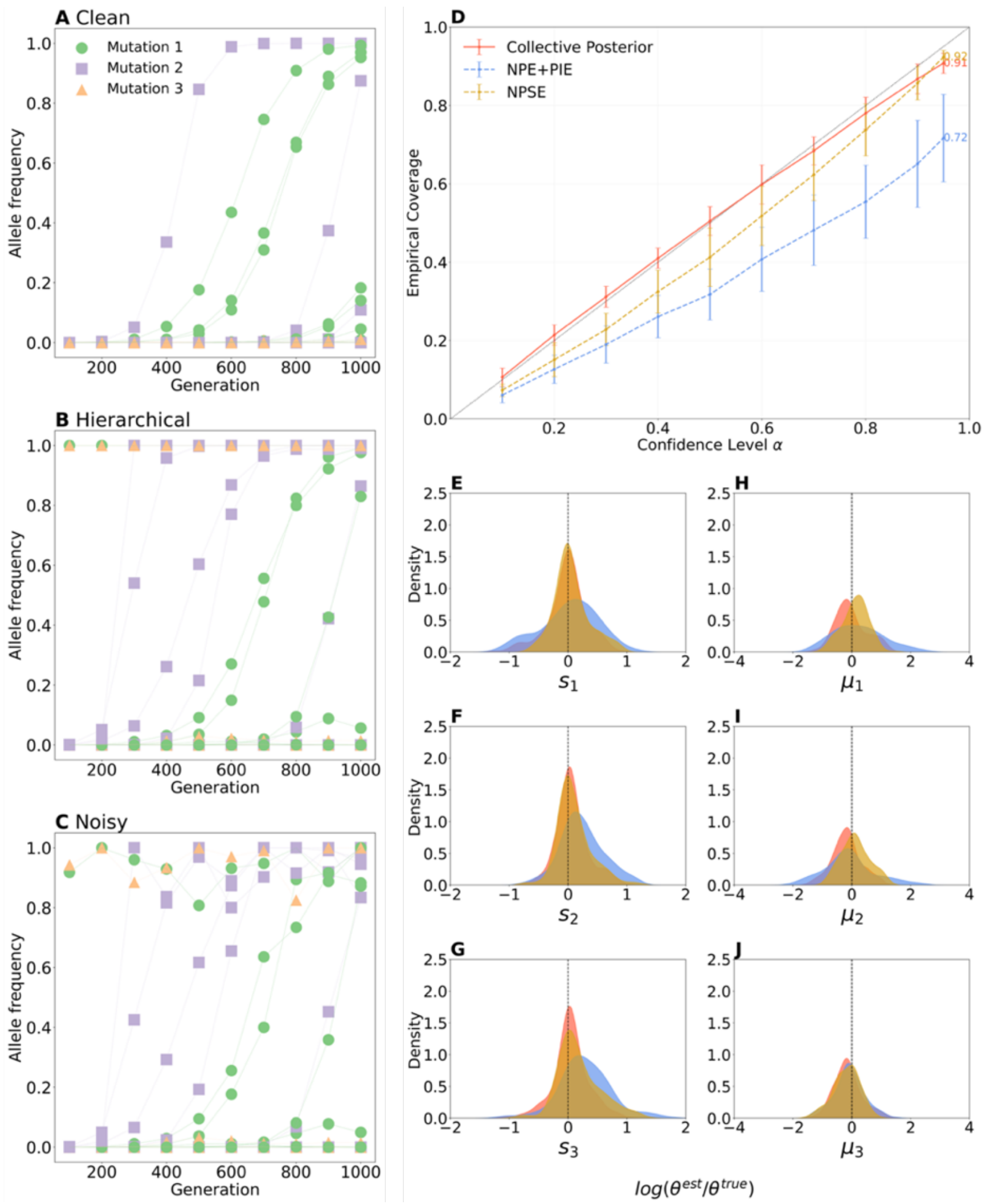

Fig 2. The collective posterior outperforms NPE+PIE and NPSE on noisy evolutionary simulations. (A-C) Synthetic replicate sets. Each test instance includes N=10 replicate time series simulated with shared parameters, θ (A, Clean). To introduce between-replicate heterogeneity, we generate seven replicates using parameters perturbed by moderate Gaussian noise and three replicates using parameters perturbed by larger Gaussian noise (B, Hierarchical). Finally, we add measurement noise to the time series (C, Noisy). **(D) Coverage.** For each nominal confidence level α, we compute the empirical coverage $Pr[\theta_i \in HDR_\alpha]$ of per-parameter highest-density regions (ideal: diagonal). The collective posterior achieved consistently better coverage (closer to the diagonal) than NPE+PIE and NPSE, indicating better calibration. **(E-J) Accuracy.** Distributions of per-parameter estimation errors (defined as the log-ratio of the posterior mean and true value). Across parameters, the collective posterior (red) is more tightly centered around zero, indicating more accurate inference.

Additionally, on empirical datasets, performance is largely judged through visual posterior predictive checks. Quantitative metrics would make the claims more convincing.

Thank you for this comment. We have added quantitative metrics for the empirical analysis in Fig 3 and described in the text (Methods, lines 363-386):

"**Evaluation metrics with empirical data.** To assess whether each method produced calibrated predictions for the empirical trajectories, we computed its *posterior predictive coverage* (Leininger and Gelfand, 2017). For each dataset and inference method, we drew parameter samples from the inferred posterior and simulated frequency trajectories with these parameter samples. At each nominal coverage level $\alpha \in \{0.1, 0.2, \ldots, 0.9, 0.95\}$, we formed central posterior predictive intervals by taking the $(1-\alpha)/2$ and $(1+\alpha)/2$ quantiles of the simulated trajectories at each generation. We then computed the fraction of empirical observations that fell within these intervals. A calibrated posterior predictive distribution should have empirical coverage close to the nominal level. We summarized calibration error as the mean absolute deviation between empirical and nominal coverage across all nominal levels. We plotted 1-MAE as a coverage score, where larger values indicate better predictive calibration. To quantify whether posterior estimates were stable, we used an *inference cycle* (Cook, Gelman and Rubin, 2006; Säilynoja *et al.*, 2026): first, we inferred the posterior distribution conditioned on the empirical data (5-8 replicates). Then, we used 10 samples from the posterior distribution to simulate a synthetic dataset of 10 replicates. We inferred a posterior distribution conditioned on the synthetic dataset and compared it to the original posterior (conditioned on the empirical data). For each parameter, we computed the *overlapping coefficient* (Weitzman's Δ, or OVL) (Pastore and Calcagnì, 2019) between the marginal posterior distributions,
$OVL(p, q) = \int \min[p(\theta), q(\theta)]\, d\theta$, where p is the original posterior marginal and q is the inference-cycle posterior marginal. The OVL ranges from 0 to 1, with 0 indicating no overlap and 1 indicating identical marginal distributions. In practice, we estimated the OVL from posterior samples by binning both sample sets on the same parameter range, normalizing the bin counts to probability masses, and summing the minimum probability mass in each bin. We report the mean OVL across parameters as a metric for inference-cycle stability, where larger values indicate greater agreement between the original and cycle posteriors."

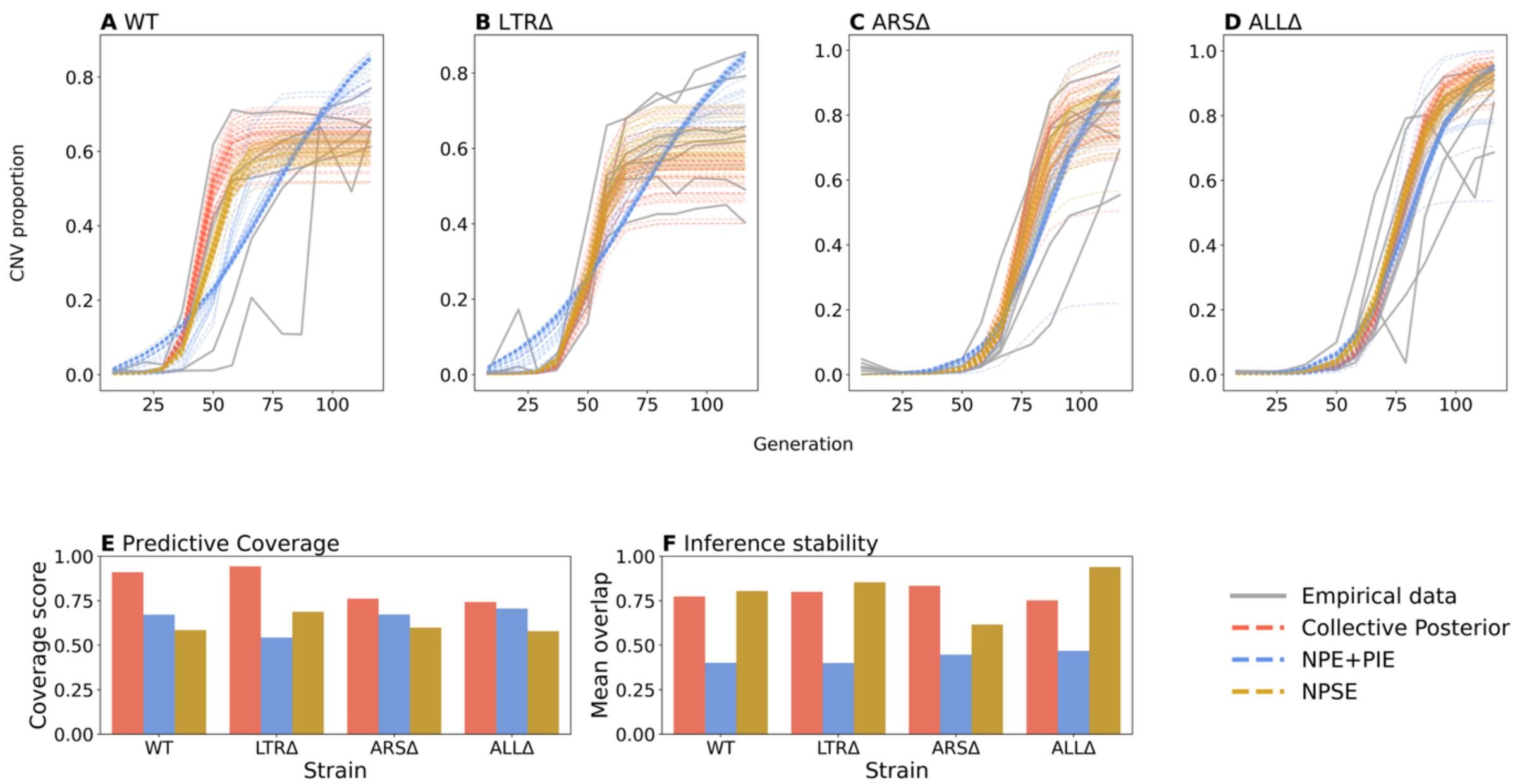

Fig 3. The collective posterior accurately captures the between-replicate variation in empirical evolutionary data. (A-D) For each of the four datasets from Chuong et al. (2025), which follow the evolution of copy number variants (CNV) in yeast populations, we simulate 50 time series using parameter sets sampled from the collective posterior with NPE (red), NPE+PIE (blue), and NPSE (yellow), and compare them to the empirical replicates (grey). The collective posterior more closely captures the observed between-replicate variability. Here, ε is estimated separately for each replicate set. **(E)** Predictive coverage, i.e., the deviation from ideal prediction-to-data calibration. **(F)** Inference stability, i.e., the overlap between the estimated posteriors (from empirical vs. synthetic data).

On the swamp sparrow reanalysis. The authors reconstruct population-specific posteriors by selecting the 100 nearest simulations from Lachlan et al.'s published output and fitting a KDE. This is a fairly rough approximation and its validity is never established. Some justification that this produces meaningful posteriors would be reassuring.

We have included a justification for the population-specific posteriors in lines 492-494 of the original text: "These replicate-specific posteriors were more concentrated and yielded lower discrepancies to the empirical summary statistics than the posterior obtained by conditioning on mean summary features across populations."

On computational efficiency. Using the prior as the SIR proposal is simple but can become very inefficient in higher dimensions. Some diagnostics like effective sample sizes would help readers assess whether this is actually practical at scale.

After diagnosing the original SIR sampler on the GLU task, we found that our method could indeed suffer from a weight collapse for high-dimensional simulators. We revised our sampling strategy and now use an adaptive SIR sampler that allows selecting a minimal effective sample size and automatically adapts the temperature accordingly. Specifically, the GLU results remain similar despite the previously lower effective sample sizes, as shown in the revised S8 Fig.

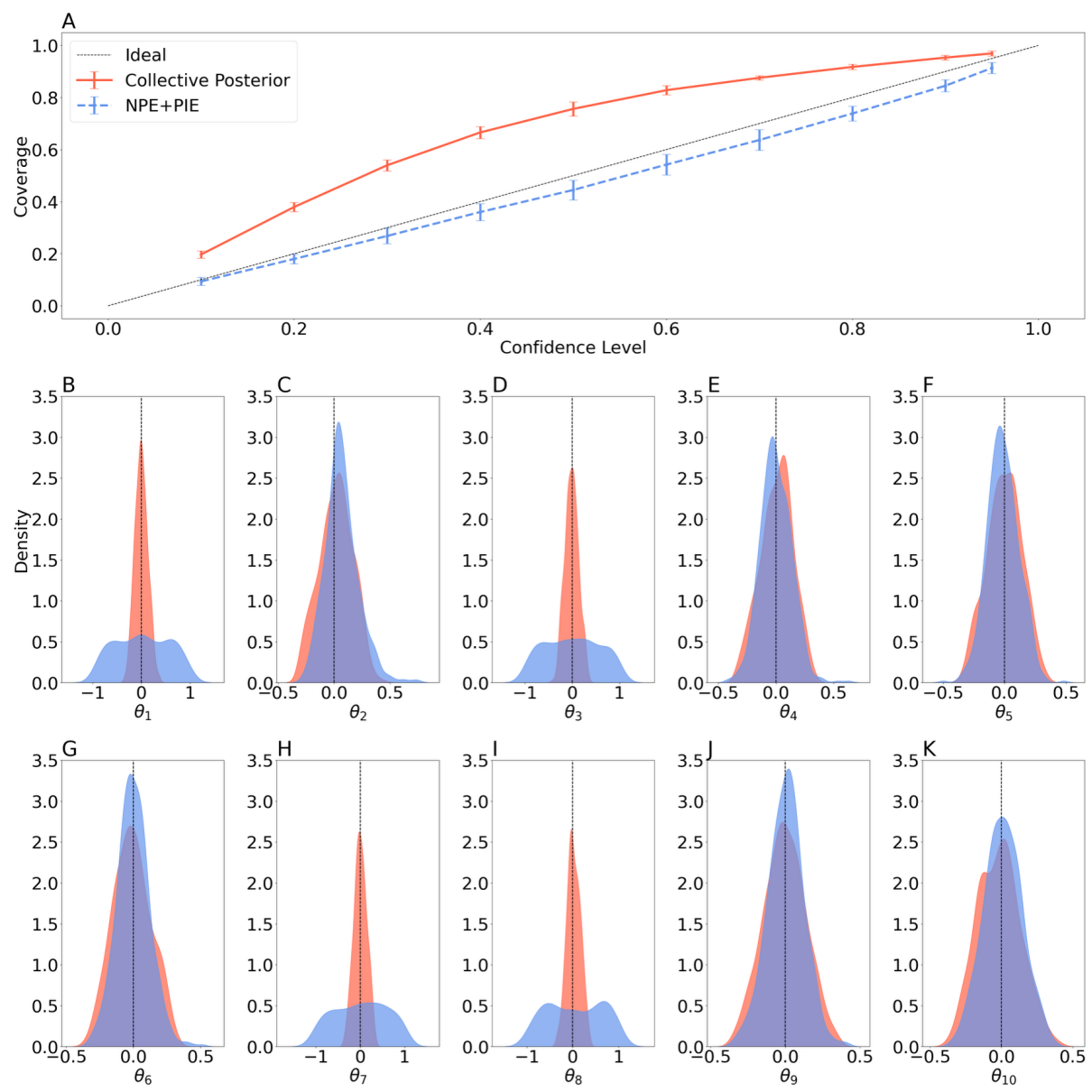

S8 Fig. The sample mean accuracy of the collective posterior and NPE + Permutation Invariant Embedding (PIE) for Gaussian Linear Uniform (GLU). Subplots show $\widehat{\theta_i} - \theta_i^{True}$ for the ten estimated parameters.

Specifically, we revised the following sections.

Methods – lines 236-248:

"**Sampling Importance Resampling (SIR).** To efficiently approximate the collective posterior without relying on sequential chains (i.e., MCMC), we used an SIR scheme (Smith and Gelfand, 1992). We used the prior distribution p(θ) as the proposal distribution to ensure robust coverage of the parameter space while not relying on the sampling efficiency of the individual posterior distribution. Importance weights were calculated as the ratio of the unnormalized robust collective posterior density to the prior density. To counteract the artificial variance reduction inherent in the product-of-experts aggregation, where multiplying independent densities shrinks the variance, we used an adaptive temperature scaling to the importance weights. This temperature was determined via

binary search to maintain a target *effective sample size* (ESS), preventing weight collapse as the number of replicates increased. Then, at resampling, we applied Gaussian jittering to the results. This vectorized approach enabled rapid, high-throughput posterior approximation. While practical and accurate, our SIR scheme is designed for high-performance approximation rather than the asymptotic sampling guarantees of MCMC."

SI – lines 66-74:

"**Adaptive temperature selection in SIR.** The unnormalized importance weights are defined as $w = e^{L(\theta)-p(\theta)}$. To prevent premature weight concentration, we introduced a temperature T. We solved for T such that the Effective Sample Size (ESS) met a minimum threshold (typically 100 of the initial 100,000 proposal pool): $ESS(T) = \frac{\left(\sum_j w_j^{\frac{1}{T}}\right)^2}{\sum_j \left(w_j^{\frac{1}{T}}\right)^2}$. Since ESS is a monotonically increasing function of temperature, we were able to use a binary search to efficiently identify the unique value that satisfies our target particle diversity.
This adaptive scaling counteracts the "peaky" geometry of the product-of-experts, ensuring that the resampled particles represent the full breadth of the collective consensus rather than a single point estimate."

All results are now based on the adaptive SIR sampler.

Overall the contribution is solid and the revisions are tractable. I look forward to seeing a revised version.

MINOR COMMENTS

There are a few rather tiny typographical and notational issues that should be corrected. For example, "stochoasticity" should read "stochasticity," and "premutation-invariant embeddings" appears to be a typographical error for "permutation-invariant embeddings."

We have carefully read the manuscript and made the following changes:
and and →and (line 227); stochoaticisity→stochasticity (line 535); dimensionaility →dimensionality (line 90); premutation →permutation (line 546); Wrigh-Fisher→Wright-Fisher (Fig 2 caption); dranaticlly→dramatically (SI line 262), underpeforms→underperforms (S16 Fig caption); a summary statistics → summary statistics (line 63); sllable → syllable (line 322); first **averaging or their** summary statistics → averaging their (line 505); the simulator ability → the simulator's ability (line 112);

Some notation could be introduced more clearly. In particular, ε is described as a minimum posterior density but the distinction between density values and probabilities is not emphasized, which may cause confusion when interpreting numerical values.

ε is a lower bound on posterior density values (not probabilities), and therefore depends on the scale of the density estimator. We have updated the text to distinguish between probabilities and densities (lines 212-223).

"**Robust posterior distribution.** Posterior distribution approximation in SBI, while accurate in the high posterior density region (HDR), tends to be uncalibrated in low-density regions, sometimes producing erratic posterior density values (Table S3). To mitigate this, we introduce a robust individual posterior distribution,

$$p_\varepsilon(\theta|x_i) = \max[\varepsilon, p(\theta|x_i)] \quad (2)$$

We combine these robust individual posterior distributions into a robust collective posterior distribution,

$$p_\varepsilon(\theta|x_1, \dots, x_n) = \frac{p(\theta)^{1-n}\Pi_i[p_\varepsilon(\theta|x_i)]}{\int p(\xi)^{1-n}\Pi_i[p_\varepsilon(\xi|x_i)]\, d\xi} \quad (3)$$

For $\varepsilon \to 0$, we have $p_\varepsilon(\theta|x_i) \to p(\theta|x_i)$ and $p_\varepsilon(\theta|x_1,\dots,x_n) \to p(\theta|x_1,\dots,x_n)$. High values of ε will focus only on high-density regions of the individual posterior distributions, overfitting to a given replicate set. An optimal choice of ε balances robustness to outliers with accurate parameter inference (Fig 1)."

The description of the Wright–Fisher simulator would benefit from slightly clearer notation for the mutation matrix and the indexing of genotype states.

We have added the following text to the SI (lines 118-122):

"**Matrix notation of the WF model.** We follow the frequencies of $X^t = \left(X^t_{wt}, X^t_{C^+}, X^t_{C^-}, X^t_B\right)^T$ for $t = 0,1,\dots,116$ generations. Each generation includes three phases: mutation, which is defined by $M = \begin{pmatrix} 1-\delta-10^{-5} & 0 & 0 & 0 \\ \delta & 1 & 0 & 0 \\ 0 & 0 & 1 & 0 \\ 10^{-5} & 0 & 0 & 1 \end{pmatrix}$, such that $X^{t'} = MX^t$; selection, which is defined by $S = \begin{pmatrix} 1 & 0 & 0 & 0 \\ 0 & 1+s & 0 & 0 \\ 0 & 0 & 1+s & 0 \\ 0 & 0 & 0 & 1.001 \end{pmatrix}$ such that $X^{t^*} = SX^{t'}$; and drift, which is defined by $X^{t+1} = \frac{1}{N_e} Multi\left(N_e, X^{t^*}\right)$."

Finally, some figure descriptions could be streamlined to help readers connect the experimental design to the results shown. For example, the panels illustrating clean, hierarchical, and noisy synthetic replicate regimes would be easier to interpret if the parameter perturbations defining each regime were briefly summarized in the figure discussion.

We have refined the figure and its caption according to the reviewers' comments, by adding the following (underlined) sentence to Fig 2 caption:

"Fig 2. The collective posterior outperforms NPE+PIE on noisy evolutionary simulations. (A-C) Synthetic replicate sets. Each test instance comprises n=10 Wright-Fisher replicate time series simulated with shared parameters, θ (A, Clean). To introduce between-replicate heterogeneity, we generate 7 replicates using parameters perturbed by moderate Gaussian noise and 3 replicates using parameters perturbed by larger Gaussian noise (B, Hierarchical). Finally, we add measurement noise to the time series (C, Noisy**). (D) Coverage.** For each nominal confidence level α, we compute the empirical coverage $\Pr[\theta_i \in HDR_\alpha]$ of per-parameter highest-density regions (ideal: diagonal). The collective posterior achieved consistently better coverage (closer to the diagonal) than NPE+PIE, indicating less overconfidence. **(E-J) Accuracy.** Distributions of per-parameter estimation errors (defined as the log-ratio of the posterior mean and true value). Across parameters, the collective posterior is more tightly centered around zero, indicating more accurate inference**.**"

Reviewer #3

Summary:
This paper studies inference from multiple conditionally independent replicates by aggregating replicate-specific posteriors into a collective posterior over shared parameters. The main issue is that NPE can behave less reliably in low-density regions, and naively multiplying per-replicate posteriors can let a single inconsistent replicate (perhaps due to a single outlier) dominate the result. The paper addresses this with a simple epsilon floor that limits the influence of any one replicate. Across synthetic SBI benchmarks and several empirical examples, the method is generally competitive with or better than NPE+PIE while requiring substantially less training time.

I found the paper interesting and potentially useful. The core idea is simple, seems easy to implement, and appealing from a practical SBI perspective. The manuscript also has good breadth of experiments and considers synthetic sbi examples as well as some interesting applications, and the reported training-cost advantage over NPE+PIE does seem meaningful.

Thank you for this positive evaluation of our manuscript.

Comments (note: fairly minor and mainly at author discretion):

1. Positioning of the contribution and relation to prior work.
As written, the abstract and introduction perhaps sometimes suggest that the posterior aggregation identity itself is novel. I might suggest sharpening the contribution statement, as the more novel contribution does seem to more specifically be the epsilon-floor robustification in this SBI with replications setting. It would also help to cite and contrast this approach with the paper "Gravitational wave populations and cosmology with neural posterior estimation" (Leyde et al., 2024) - an SBI approach them seems to also be considering the "Eq 1." scenario, and (optionally) briefly situate the method relative to alternative approaches that could have been done, such as weighted or tempered PoE variants (in contrast to the epsilon flooring approach).

Thank you for this helpful suggestion. We agree that the posterior aggregation identity itself follows directly from standard conditional-independence assumptions and is not our primary contribution. We revised the Abstract and Introduction to clarify that our main contribution is the robust ε-floor aggregation scheme for simulation-based inference from noisy multiple-replicate datasets. Specifically, we have added the following text to the introduction (lines 125-130):
"Hierarchical posterior aggregation under conditional independence has also been explored in cosmology. For example, Leyde et al. (Leyde *et al.*, 2024) applied neural posterior estimation to hierarchical gravitational-wave population inference, combining information from multiple independent observations through sequential recombination of sub-population analyses. In contrast, our work focuses specifically on robustness to replicate heterogeneity, outliers, and posterior density pathologies in noisy biological replicate datasets."

2. Sampling diagnostics for the SIR step.

The main results rely on prior-proposal SIR with temperature scaling and Gaussian jitter. Since the practical efficiency of this step depends on how concentrated the importance weights become, I think the paper could report a simple sampling diagnostic, such as the ESS of the resampling weights. In particular for the 10-dimensional GLU benchmark, just to check weight degeneracy isn't an issue here.

After diagnosing the original SIR sampler we found that it indeed suffered from a collapse issue for high-dimensional simulators like the GLU. We revised it and now use an **adaptive SIR sampler** that allows selecting a minimal effective sample size and automatically adapts the temperature accordingly. This improves the practical use of our method. The GLU results remain similar despite the previously lower effective sample sizes, as displayed in the revised S8 Fig. Specifically, we revised the following sections:

Methods – lines 236-248:

"**Sampling Importance Resampling (SIR).** To efficiently approximate the collective posterior without relying on sequential chains (i.e., MCMC), we used an SIR scheme (Smith and Gelfand, 1992). We used the prior distribution p(θ) as the proposal distribution to ensure robust coverage of the parameter space while not relying on the sampling efficiency of the individual posterior distribution. Importance weights were calculated as the ratio of the unnormalized robust collective posterior density to the prior density. To counteract the artificial variance reduction inherent in the product-of-experts aggregation, where multiplying independent densities shrinks the variance, we used an adaptive temperature scaling to the importance weights. This temperature was determined via binary search to maintain a target *effective sample size* (ESS), preventing weight collapse as the number of replicates increased. Then, at resampling, we applied Gaussian jittering to the results. This vectorized approach enabled rapid, high-throughput posterior approximation. While practical and accurate, our SIR scheme is designed for high-performance approximation rather than the asymptotic sampling guarantees of MCMC."

SI – lines 66-74:

"**Adaptive temperature selection in SIR.** The unnormalized importance weights are defined as $w = e^{L(\theta)-p(\theta)}$. To prevent premature weight concentration, we introduced a temperature T. We solved for T such that the Effective Sample Size (ESS) met a minimum threshold (typically 100 of the initial 100,000 proposal pool): $ESS(T) = \frac{\left(\sum_j w_j^{\frac{1}{T}}\right)^2}{\sum_j \left(w_j^{\frac{1}{T}}\right)^2}$. Since ESS is a monotonically increasing function of temperature, we were able to use a binary search to efficiently identify the unique value that satisfies our target particle diversity.

This adaptive scaling counteracts the "peaky" geometry of the product-of-experts, ensuring that the resampled particles represent the full breadth of the collective consensus rather than a single point estimate."

All results are now based on the adaptive SIR sampler.

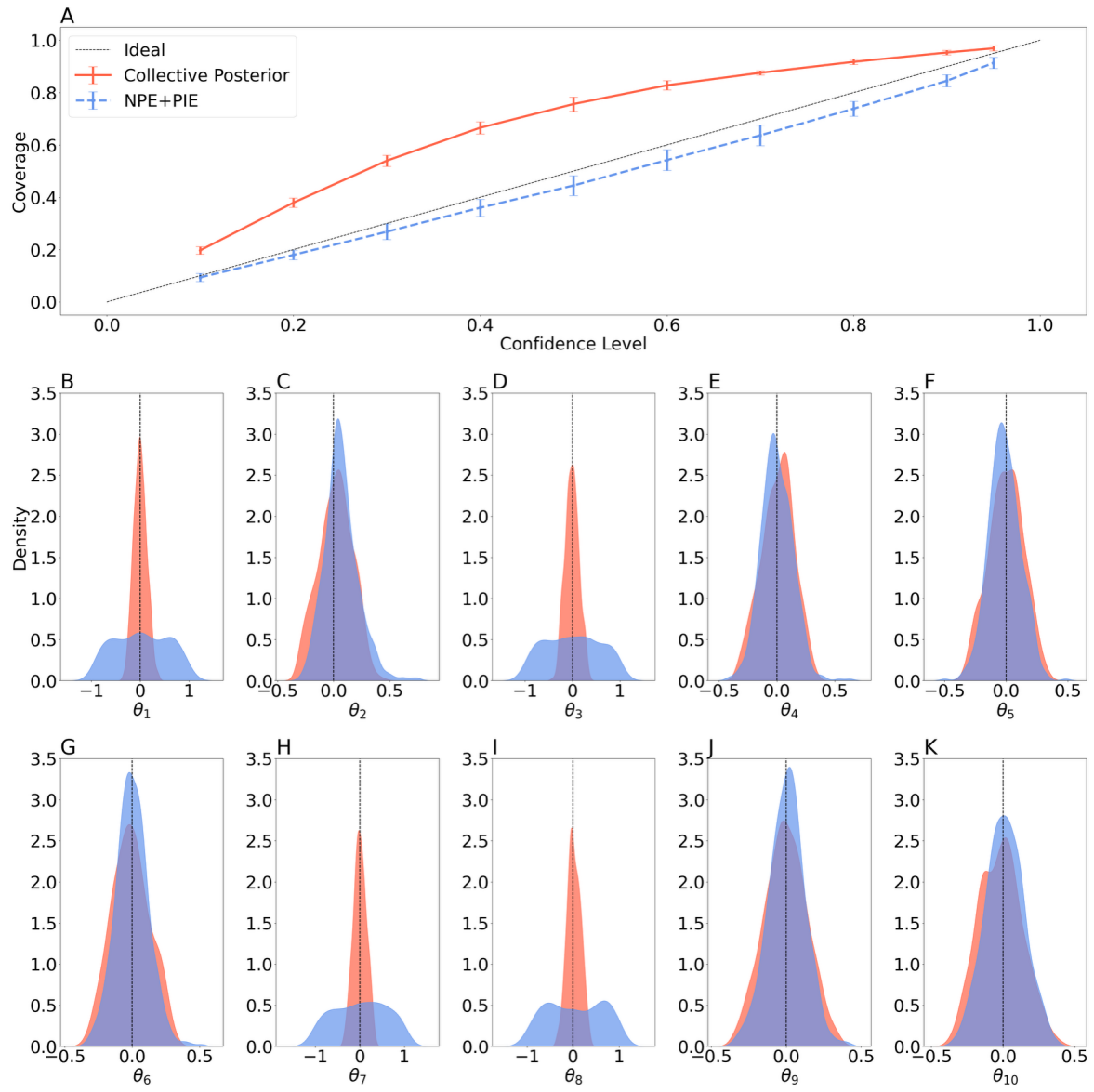

S8 Fig. The sample mean accuracy of the collective posterior and NPE + Permutation Invariant Embedding (PIE) for Gaussian Linear Uniform (GLU). Subplots show $\widehat{\theta_i} - \theta_i^{True}$ for the ten estimated parameters.

3. Framing as robustness to misspecification.
The motivating failure mode here of replicates differing more than the simulator predicts because of batch effects, biological heterogeneity, or simulation-to-reality gap, as noted in the paper, could be viewed as a model misspecification problem, I think this could be made a bit more explicit and could connect with related work in the SBI literature. Perhaps could optionally add a couple sentences/clarifications, situation this epsilon-floor approach in that context might help readers evaluate using this approach vs other robust SBI approaches (e.g., potentially there is a connection here with generalised Bayesian inference).

We agree that the motivating failure mode in our setting — where empirical replicates exhibit greater variability than predicted by the simulator due to batch effects, biological heterogeneity, or simulation-to-reality gap — can be viewed as a form of model misspecification. We now make this framing more explicit in the Introduction and Discussion.
In particular, we clarify that the ε-floor method can be interpreted as a robustness mechanism that limits the influence of replicates whose inferred posterior densities become unrealistically small under the assumed model. We additionally added a brief discussion connecting our approach to broader robust Bayesian and simulation-based inference literature, including generalized Bayesian inference approaches that modify or temper likelihood contributions under model misspecification. We emphasize that our approach differs in operating directly at the level of replicate-specific posterior aggregation, while remaining compatible with arbitrary amortized posterior estimators and requiring minimal modification of existing SBI pipelines.

Specifically, we have added the following text to the Discussion (lines 547-565):

"In our setting, the motivating failure mode occurs when empirical replicates differ more than predicted by the simulator due to batch effects, biological heterogeneity, or the simulation-to-reality gap. This discrepancy can be viewed as a form of model misspecification. Under misspecification, standard product aggregation, which conditions on all replicates assuming they are independent and identically distributed (i.i.d.), can become highly unstable. This instability arises because posterior inference for a single replicate focuses heavily on accurately estimating high-density regions, leaving tail estimates potentially inaccurate and noisy. While the distinction between different extreme low densities is practically irrelevant for a single replicate, it becomes catastrophic during product aggregation when replicates disagree on where the high-density regions lie. Because density estimators cannot reliably distinguish between these low-density values, a single conflicting replicate can effectively zero out parameter regions strongly supported by the rest. The ε-floor regularization introduced here directly resolves this by acting as a robustness mechanism, bounding the influence of these unreliable, low-density tail contributions during aggregation. This perspective connects our approach to broader robust Bayesian and simulation-based inference frameworks, including generalized Bayesian inference approaches that temper or modify likelihood contributions under model misspecification (Bissiri, Holmes and Walker, 2016; Miller and Dunson, 2019). In contrast to these methods, our approach operates directly at the level of replicate-specific posterior aggregation and remains compatible with any posterior estimators and existing SBI methods without any modification."

Spelling and grammar

The manuscript would benefit from a careful proofread. Some errors include "and and" (p. 10), "stochoasticity" (p. 12), "sllable" (p. 14), "tighly" (p. 17), "dimensionaility" (p. 20), "premutation-invariant" (p. 21), and "Wrigh-Fisher" in the Figure 2 caption, as well as "dranatically" and "underpeforms" in the supplementary Figure S16 caption.

We have carefully read the manuscript and made the following changes:

and and →and (line 227); stochoaticisity→stochasticity (line 535); dimensionaility →dimensionality (line 90); premutation →permutation (line 546); Wrigh-Fisher→Wright-Fisher (Fig 2 caption); dranaticlly→dramatically (SI line 262), underpeforms→underperforms (S16 Fig caption); a summary statistics → summary statistics (line 63); sllable → syllable (line 322); first **averaging or their** summary statistics → averaging their (line 505); the simulator ability → the simulator's ability (line 112);

References

Avecilla, G. *et al.* (2022) "Neural networks enable efficient and accurate simulation-based inference of evolutionary parameters from adaptation dynamics," *PLOS Biology*, 20(5), p. e3001633. Available at: https://doi.org/10.1371/journal.pbio.3001633.

Caspi, I. *et al.* (2023) "Mutation rate, selection, and epistasis inferred from RNA virus haplotypes via neural posterior estimation," *Virus Evolution*, 9(1), p. vead033.

Chuong, J.N. *et al.* (2025) "Template switching during DNA replication is a prevalent source of adaptive gene amplification," *eLife*, 13, p. RP98934.

Cook, S.R., Gelman, A. and Rubin, D.B. (2006) "Validation of Software for Bayesian Models Using Posterior Quantiles," *Journal of Computational and Graphical Statistics*, 15(3), pp. 675–692. Available at: https://doi.org/10.1198/106186006X136976.

De, T. *et al.* (2025) "Segmental copy number amplifications are stable in the absence of selection," *bioRxiv*, p. 2025.07.21.665951. Available at: https://doi.org/10.1101/2025.07.21.665951.

Lachlan, R.F., Ratmann, O. and Nowicki, S. (2018) "Cultural conformity generates extremely stable traditions in bird song," *Nature Communications*, 9(1), p. 2417. Available at: https://doi.org/10.1038/s41467-018-04728-1.

Leininger, T.J. and Gelfand, A.E. (2017) "Bayesian Inference and Model Assessment for Spatial Point Patterns Using Posterior Predictive Samples," *Bayesian Analysis*, 12(1), pp. 1–30. Available at: https://doi.org/10.1214/15-BA985.

Pastore, M. and Calcagnì, A. (2019) "Measuring Distribution Similarities Between Samples: A Distribution-Free Overlapping Index," *Frontiers in Psychology*, 10. Available at: https://doi.org/10.3389/fpsyg.2019.01089.

Säilynoja, T. *et al.* (2026) "Posterior SBC: simulation-based calibration checking conditional on data," *Statistics and Computing*, 36(2), p. 78. Available at: https://doi.org/10.1007/s11222-026-10825-9.

Smith, A.F.M. and Gelfand, A.E. (1992) "Bayesian Statistics without Tears: A Sampling–Resampling Perspective," *The American Statistician*, 46(2), pp. 84–88. Available at: https://doi.org/10.1080/00031305.1992.10475856.
