# Supplementary material for: Collective posterior inference from highly variable empirical replicates
Source: PLoS Comput Biol. 2026 Aug 11;22(8):e1014534. doi: 10.1371/journal.pcbi.1014534 (PMC13475975; doi:10.1371/journal.pcbi.1014534)
Supplement: Supplementary file 2 [file pcbi.1014534.s024.docx]

**Collective Posterior Inference from Highly Variable Empirical Replicates**

**Supporting Information**

Nadav Ben Nun^1,2^, Saharon Rosset^3^, David Gresham^4^, Yoav Ram^1,2,*^

1. School of Zoology, Faculty of Life Sciences, Tel Aviv University, Tel Aviv, Israel

2. Edmond J. Safra Center for Bioinformatics, Tel Aviv University, Tel Aviv, Israel

3. Department of Statistics, Tel Aviv University, Tel Aviv, Israel

4. Department of Biology, Center for Genomics and Systems Biology, New York University, New York, United States

* Corresponding author: yoavram@tauex.tau.ac.il

**Full Derivation of the Collective Posterior Distribution**

Let $x_{1},\ldots,x_{n}$be empirical observations such that $x_{i}\sim p\left( x|\theta\right).$
Assuming we have already estimated $p\left( \theta| x_{i} \right)$ using NPE, we wish to estimate $p\left( \theta| x_{1},\ldots,x_{n} \right)$.

Using Bayes’ theorem,

$$\left( 1 \right) p\left( \theta| x_{1},\ldots,x_{n} \right)=\frac{p\left( x_{1},\ldots,x_{n} \right|\theta)p(\theta)}{p\left( x_{1},\ldots x_{n} \right)} ,$$

Assuming $x_{i},x_{j}$ are independent given $\theta$, i.e., $p\left( x_{i},x_{j} | \theta\right)=p\left( x_{i} | \theta\right)p\left( x_{j} | \theta\right)$, we get

$$\left( 2 \right) p\left( \theta| x_{1},\ldots,x_{n} \right)=\frac{\Pi_{i}\left[ p\left( x_{i} | \theta\right) \right]}{p\left( x_{1},\ldots x_{n} \right)}p\left( \theta\right) ,$$

Using Bayes’ rule again,

$$\left( 3 \right) p\left( \theta| x_{1},\ldots,x_{n} \right)=\frac{\Pi_{i}\left[ p\left( \theta| x_{i} \right)p\left( x_{i} \right) \right]}{p\left( x_{1},\ldots x_{n} \right)}p\left( \theta\right)^{1-n} .$$

Next, recalling the law of total probability

$$p\left( x_{1},\ldots,x_{n} \right)=\int P\left( x_{1},\ldots,x_{n} | \xi\right)p\left( \xi\right)d\xi.$$

Using Bayes’ rule,

$$p\left( x_{1},\ldots,x_{n} \right)=\int\frac{p\left( \xi| x_{1},\ldots,x_{n} \right)p\left( x_{1},\ldots,x_{n} \right)}{p\left( \xi\right)} p\left( \xi\right)d\xi.$$

As $p\left( x_{1},\ldots,x_{n} \right)$ is positive and independent of $\xi$, we can take it out of the integral and cancel it out from both sides,

$$\left( 4 \right) \int P\left( \xi| x_{1},\ldots,x_{n} \right)d\xi=1 .$$

Using Bayes’ rule in (4),

$$\int\frac{\Pi_{i}\left[ p\left( \xi| x_{i} \right)p\left( x_{i} \right) \right]}{p\left( x_{1},\ldots x_{n} \right)}p\left( \xi\right)^{1-n}d\xi=1 .$$

Using commutativity of multiplication,

$$\left( 5 \right) \int\frac{\Pi_{j}\left[ p\left( x_{j} \right) \right]}{p\left( x_{1},\ldots x_{n} \right)}p\left( \xi\right)^{1-n}\Pi_{i}\left[ p\left( \xi| x_{i} \right) \right]d\xi=1 ,$$

Where $p(\xi)$, the prior, is known to us, and $p(\xi|x_{i})$, the individual posteriors, are estimated by a density estimator. Therefore, only the marginal likelihoods $p(x_{i})$ and $p(x_{1},\ldots x_{n})$ are unknown, yet positive and independent of $\xi$.

We can therefore consider $\frac{\Pi_{j}p\left( x_{j} \right)}{p\left( x_{1},\ldots x_{n} \right)}=C$ and get

$$\int C\cdot p\left( \xi\right)^{1-n}\Pi_{i}\left[ p\left( \xi| x \right) \right]d\xi=1 ,$$

$$\left( 6 \right) C=\frac{1}{\int p\left( \xi\right)^{1-n}\Pi_{i}\left[ p\left( \xi| x_{i} \right) \right]d\xi} .$$

Then, plugging C in (3) will result in the collective posterior distribution as in eq. 1,

$$\boldsymbol{p}\left( \boldsymbol{\theta} | \boldsymbol{x}_{\boldsymbol{1}}\boldsymbol{,\ldots,}\boldsymbol{x}_{\boldsymbol{n}} \right)\boldsymbol{=}\frac{{\boldsymbol{p}\left( \boldsymbol{\theta} \right)^{\boldsymbol{1-n}}\boldsymbol{\Pi}}_{\boldsymbol{i}}\left[ \boldsymbol{p}\left( \boldsymbol{\theta} | \boldsymbol{x}_{\boldsymbol{i}} \right) \right]}{\boldsymbol{\int p}\left( \boldsymbol{\xi} \right)^{\boldsymbol{1-n}}\boldsymbol{\Pi}_{\boldsymbol{i}}\left[ \boldsymbol{p}\left( \boldsymbol{\xi} | \boldsymbol{x}_{\boldsymbol{i}} \right) \right]\boldsymbol{d\xi}}\boldsymbol{.}$$

**Additional Sampling Strategies**

**Adaptive temperature selection in SIR.** The unnormalized importance weights are defined as $w=e^{L\left( \theta\right)-p(\theta)}$. To maintain a healthy particle diversity, we introduced a temperature $T$. We solved for $T$ such that the Effective Sample Size (ESS) met a minimum threshold (typically 100 of the initial 100,000 proposal pool): $ESS\left( T \right)=\frac{\left( \sum_{j} w_{j}^{\frac{1}{T}} \right)^{2}}{\sum_{j} \left( w_{j}^{\frac{1}{T}} \right)^{2}}$ . Since ESS is a monotonically increasing function of temperature, we were able to use a binary search to efficiently identify the unique  that satisfies our target particle diversity.
This adaptive scaling counteracts the "peaky" geometry of the product-of-experts, ensuring that the resampled particles represent the full breadth of the collective consensus rather than a single point estimate.

**Markov Chain Monte Carlo (MCMC).** As a robust alternative for exploring complex local landscapes, we implemented a Metropolis–Hastings sampler initialized from high-probability modes. We first generated a large pool of candidates from the prior and evaluated their unnormalized posterior probabilities. The 50 candidates with the highest log-probabilities were selected to initialize 50 independent Markov chains. Each chain proceeded with Metropolis–Hastings updates to locally explore the posterior mass, ensuring adequate coverage of the collective posterior's peaks even in cases where the global proposal distribution of SIR might be inefficient.

**Rejection Sampling.** We also implemented exact rejection sampling. This required first approximating the normalizing constant $\frac{1}{C}$ (the denominator of Eq. 1) via Monte Carlo integration using $N_{C}={10}^{5}$ prior samples, and averaged across 10 independent approximations. Once the constant was estimated, samples were drawn from the prior and accepted or rejected based on the ratio of the collective posterior probability to the proposal. While asymptotically exact, this method proved computationally prohibitive for high-dimensional or highly peaked posteriors due to vanishing acceptance rates.

**Hyperparameter tuning.** Sampling with MCMC or rejection sampling introduces two additional hyperparameters: $N_{samples}$, the number of initial candidate samples to keep for MCMC sampling; and $N_{C}$, the number of prior samples to evaluate the denominator of eq. (1) for sampling from the posterior distributions.. For $N_{\text{samples}}$, we set a default of 50, corresponding to the 50 candidate points with the highest log-probability under the estimated normalization constant $\frac{1}{C}$. These candidates serve as initialization points for independent MCMC chains, ensuring sufficient exploration of multiple posterior modes while avoiding over-concentration in a single region of high probability. For comparison, *sbi* typically employs 20 independent MCMC chains, following the recommendations of Neural Likelihood Estimation (2). We extended this number since a collective posterior distribution can potentially have a larger number of modalities, therefore requiring a broader inspection of the support.
Finally, sampling from the collective distribution requires a one-time computation of *C*, with computation time scaling linearly with $N_{C}$, increasing from a few seconds for $N_{C}={10}^{5}$ to about a minute with $N_{C}={10}^{6}$ on a 48-core CPU.

**Wright-Fisher model (3)**

**Matrix notation of the WF model.** We follow the frequencies of $X^{t}=\left( X_{wt}^{t}, X_{C^{+}}^{t},X_{C^{-}}^{t}, X_{B}^{t} \right)^{T}$ for $t=0,1,\ldots,116$ generations. Each generation includes three phases: mutation, which is defined by $M=\left( \begin{matrix} 1-\delta-{10}^{-5} & 0 & 0 & 0 \\ \delta& 1 & 0 & 0 \\ 0 & 0 & 1 & 0 \\ {10}^{-5} & 0 & 0 & 1 \end{matrix} \right)$, such that ${X^{t}}^{'}=MX^{t}$; selection, which is defined by $S=\left( \begin{matrix} 1 & 0 & 0 & 0 \\ 0 & 1+s & 0 & 0 \\ 0 & 0 & 1+s & 0 \\ 0 & 0 & 0 & 1.001 \end{matrix} \right)$ such that ${X^{t}}^{*}=MX^{t}'$; and drift, which is defined by $X^{t+1} =\frac{1}{N_{e}}Multi\left( N_{e}, {X^{t}}^{*} \right)$.

**Score-based methods for SBI from multiple observations**

Neural posterior score estimation and diffusion models (4–6) have shown promise for simulation-based inference from i.i.d replicates. We therefore compared NPSE with the Gaussian sampler (5) to collective posterior inference with standard NPE on both the synthetic and empirical data.

To train NPE, we used Masked Autoregressive Flow (MAF) (7) as the neural density estimator. MAF is recommended mostly for its training time, while considered subpar in terms of accuracy. Both Geffner et al. and Linhart et al. showed that their methods outperform NPE with MAF on several benchmarks, meaning that MAF is not the optimal replicate-specific posterior estimator. Here, we show that our collective posterior inference, with MAF as its neural density estimator, slightly outperforms NPSE in the noisy biological setting (S6 Fig). Importantly, our method is orders of magnitude faster at inference, with similar training times (Table S1).

Arruda et al. (6) introduced a score-based method for efficiently estimating a posterior distribution from a large number of i.i.d. replicates and simulators with many parameters. While the method is promising, it requires significant hyperparameter tuning, which is non-trivial for general users, and is limited to a specific (score-based) density estimator. We recommend attempting their method, especially for massive datasets or parameter-heavy simulators. However, our collective posterior inference is agnostic to the density estimator and is essentially free of hyperparameter tuning, making it extremely applicable to new problems.

**An Additional (unrealistic) Test Case – Genome Rearrangement**

We applied our method to a recent genome rearrangement model (9), which models genome evolution along a phylogenetic tree due to rearrangement events: inversion, a reversal of a block of genes in its place, with a rate $R_{in}$; translocation, a relocation of a block of genes, with a rate $R_{tr}$; fusion, merging of two chromosomes to one, with a rate $R_{fu}$; fission, splitting of one chromosome to two, with a rate $R_{fi}$. Block sizes (for inversion and translocation) follow a trimmed Zipfian distribution with an estimated exponent parameter $a$ and a fixed maximal value $m_{zip}=20$, such that $f\left( a,m_{zip} \right)=\frac{k^{-a}}{\Sigma_{i=1}^{m_{zip}}i^{-a}}$. The model output is a set of genomes, one for each of the taxa on the terminal nodes of the phylogenetic tree. This model has high variance among simulations with the same parameter values, and we further increase this variance by considering a very large number of chromosomes in the ancestral genome, 90 (S10 Fig). Moshe et. al validated their simulation-based inference method on synthetic data and applied it to a set of eighteen *Candida* species (10). Here, we simulated ten independent synthetic observations using their estimated parameters. Then, we estimated the individual posteriors using rejection-ABC and aggregated them to a collective posterior distribution.

We generated ten synthetic observations using the parameters estimated by Moshe et al. (2022). Using rejection-ABC inference for individual posterior distributions, the collective posterior distribution concentrated around the true parameters (S11 Fig). Notably, the collective posterior had the highest density for the true value of all parameters and was bi-modal for $a$ and $R_{fu}$ (S11 Fig). This bimodality reflects the diverse individual posterior distributions.
While the simulator applies to empirical data, it is highly unrealistic to obtain multiple independent observations with the same phylogenetic tree. We chose to include this example to show the applicability of the collective posterior to additional types of biological simulators.

**Single-locus Wright–Fisher task**

We evaluated the collective posterior distribution and NPE+PIE on a Wright–Fisher simulator tracking a single locus with one beneficial mutation. The model follows the same evolutionary process as the three-locus setup (mutation → selection → drift), but observations consist of 20 samples recorded every 10 generations. In this setting, we infer the selection coefficient $s$, mutation rate $\delta$, and effective population size $N_{e}$.
Although this simulator is computationally efficient, it lacks inherent variance that would necessitate collective inference methods (S15 Fig). When tested on ten replicate observations, NPE+PIE showed a clear advantage in estimating the selection coefficient, whereas the collective posterior was more accurate for $N_{e}$ (S16 Fig). However, after perturbing the parameters with Gaussian noise $N(0,0.2)$, the collective posterior outperformed NPE+PIE across all parameters (S17 Fig).

**Floor-Raising Ensembles**

If we view the collective posterior distribution as a robust posterior aggregator, we can apply it to single observations in the context of ensembles. Much like experimental outliers, one or more of the models in the ensembles could be biased or overconfident. This will either skew or overspread the posterior distribution. In contrast, using the collective posterior could produce an ensemble that concentrates on the intersection of high-density regions (S18 Fig).

**Dependence on ε and N replicates**

To further validate the *Collective Posterior*, we ran the evolutionary benchmark analysis for smaller replicate numbers: 3, 5, or 7 replicates. First, we reran the ε estimation for every single dataset to test the variance across estimations. Estimating ε using the 95^th^ percentile yielded stable results that resemble the single $\varepsilon=e^{-10}$ global estimation (S19 Fig).
Then, we ran the evolutionary benchmark task for all replicate numbers (3, 5, 7, and 10), and for varying epsilon values ($e^{-1000}\leq\varepsilon\leq e^{10}$, S20 Fig). The results show that ε highly affects both the accuracy and the coverage of our method far more than the number of replicates. While increasing the number of replicates slightly improves accuracy and does not affect coverage, ε has an optimal value for accuracy. In addition, while there is no clear optimal ε when we consider both accuracy and coverage, our estimated ε leads to optimal accuracy and a competitive coverage performance (S20 Fig).

**References – Supporting Information**

1.	Cranmer K, Brehmer J, Louppe G. The frontier of simulation-based inference. Proc Natl Acad Sci. 2020 Dec;117(48):30055–62.

2.	Papamakarios G, Sterratt D, Murray I. Sequential Neural Likelihood: Fast Likelihood-free Inference with Autoregressive Flows. In: Proceedings of the Twenty-Second International Conference on Artificial Intelligence and Statistics [Internet]. PMLR; 2019 [cited 2025 Feb 9]. p. 837–48. Available from: https://proceedings.mlr.press/v89/papamakarios19a.html

3.	Chuong JN, Nun NB, Suresh I, Matthews JC, De T, Avecilla G, et al. Template switching during DNA replication is a prevalent source of adaptive gene amplification. eLife. 2025;13:RP98934.

4.	Geffner T, Papamakarios G, Mnih A. Compositional Score Modeling for Simulation-Based Inference. In: Proceedings of the 40th International Conference on Machine Learning [Internet]. PMLR; 2023 [cited 2025 Feb 9]. p. 11098–116. Available from: https://proceedings.mlr.press/v202/geffner23a.html

5.	Linhart J, Cardoso GV, Gramfort A, Corff SL, Rodrigues PLC. Diffusion posterior sampling for simulation-based inference in tall data settings [Internet]. arXiv; 2024 [cited 2025 Feb 9]. Available from: http://arxiv.org/abs/2404.07593

6.	Arruda J, Pandey V, Sherry C, Barroso M, Intes X, Hasenauer J, et al. Compositional amortized inference for large-scale hierarchical Bayesian models [Internet]. arXiv; 2025 [cited 2025 Dec 9]. Available from: http://arxiv.org/abs/2505.14429

7.	Papamakarios G, Pavlakou T, Murray I. Masked Autoregressive Flow for Density Estimation [Internet]. arXiv; 2018 [cited 2025 Nov 10]. Available from: http://arxiv.org/abs/1705.07057

8.	Lueckmann JM, Boelts J, Greenberg D, Goncalves P, Macke J. Benchmarking Simulation-Based Inference. In: Proceedings of The 24th International Conference on Artificial Intelligence and Statistics [Internet]. PMLR; 2021 [cited 2025 Feb 9]. p. 343–51. Available from: https://proceedings.mlr.press/v130/lueckmann21a.html

9.	Moshe A, Wygoda E, Ecker N, Loewenthal G, Avram O, Israeli O, et al. An approximate Bayesian computation approach for modeling genome rearrangements. Mol Biol Evol. 2022;39(11):msac231.

10.	Maguire SL, ÓhÉigeartaigh SS, Byrne KP, Schröder MS, O’Gaora P, Wolfe KH, et al. Comparative Genome Analysis and Gene Finding in Candida Species Using CGOB. Mol Biol Evol. 2013 Jun;30(6):1281–91.
