# Supplementary material for: Collective posterior inference from highly variable empirical replicates
Source: PLoS Comput Biol. 2026 Aug 11;22(8):e1014534. doi: 10.1371/journal.pcbi.1014534 (PMC13475975; doi:10.1371/journal.pcbi.1014534)
Supplement: Peer review history

# Peer review: Decision Letter 0

**DOI:** 10.1371/journal.pcbi.1014534.r001

**Authors:** Tobias Bollenbach, Raymond Louie

16 Apr 2026

PCOMPBIOL-D-26-00248

Collective Posterior Inference from Highly Variable Empirical Replicates

PLOS Computational Biology

Dear Dr. Ram,

Thank you for submitting your manuscript to PLOS Computational Biology. After careful consideration, we feel that it has merit but does not fully meet PLOS Computational Biology's publication criteria as it currently stands. Therefore, we invite you to submit a revised version of the manuscript that addresses the points raised during the review process.

Please submit your revised manuscript by Jun 16 2026 11:59PM. If you will need more time than this to complete your revisions, please reply to this message or contact the journal office at ploscompbiol@plos.org. When you're ready to submit your revision, log on to https://www.editorialmanager.com/pcompbiol/ and select the 'Submissions Needing Revision' folder to locate your manuscript file.

Please include the following items when submitting your revised manuscript:

* A letter that responds to each point raised by the editor and reviewer(s). You should upload this letter as a separate file labeled 'Response to Reviewers'. This file does not need to include responses to formatting updates and technical items listed in the 'Journal Requirements' section below.

* A marked-up copy of your manuscript that highlights changes made to the original version. You should upload this as a separate file labeled 'Revised Manuscript with Track Changes'.

* An unmarked version of your revised paper without tracked changes. You should upload this as a separate file labeled 'Manuscript'.

If you would like to make changes to your financial disclosure, competing interests statement, or data availability statement, please make these updates within the submission form at the time of resubmission. Guidelines for resubmitting your figure files are available below the reviewer comments at the end of this letter

We look forward to receiving your revised manuscript.

Kind regards,

Raymond Louie

Academic Editor

PLOS Computational Biology

Tobias Bollenbach

Section Editor

PLOS Computational Biology

**Additional Editor Comments:**

I apologize for the lateness of the responise. It has been a journey finding reviews for the paper.

Overall, the reviewers found the manuscript interesting, timely, and potentially impactful, praising the simplicity and practical appeal of the core idea for aggregating replicate-level posteriors in simulation-based inference. The reviewers raise some important issues which need to be addressed, including clarifying assumptions underlying the method, further benchmarking, and efficiency of using the prior.

**Journal Requirements:**

1) We ask that a manuscript source file is provided at Revision. Please upload your manuscript file as a .doc, .docx, .rtf or .tex. If you are providing a .tex file, please upload it under the item type u2018LaTeX Source Fileu2019 and leave your .pdf version as the item type u2018Manuscriptu2019.

2) Please upload all main figures as separate Figure files in .tif or .eps format. For more information about how to convert and format your figure files please see our guidelines:

https://journals.plos.org/ploscompbiol/s/figures

3) We have noticed that you have uploaded Supporting Information files, but you have not included a list of legends. Please add a full list of legends for your Supporting Information files after the references list.

**Reviewers' comments:**

Reviewer's Responses to Questions

**Comments to the Authors:**

**Please note here if the review is uploaded as an attachment.**

Reviewer #1: Overall comments

1. I would like to understand better the dependence on both epsilon and N replicates.

2. In Fig 5, the method seems to introduce peaks that aren't in the individual posteriors. This should either be explained or investigated further.

3. While the github repo is very close to being great, it really needs some more work to make installation easier.

I may be misunderstanding, but the adaptive heuristic method seems to just shift the assumed hyperparameter from epsilon to the percentile value.

This isn't necessarily bad, but then it seems like there should be more discussion of the chosen percentiles.

The interplay of the percentile choice and N replicates seems especially troublesome.

Since the whole point of the method is application to relatively small N, it seems that small changes in epsilon/percentile could make a huge difference in results (by essentially excluding a large fraction of the data).

It seems like choosing the percentile is tantamount to deciding how many outliers to allow, which of course would be bad since we don't know that ahead of time.

Even if I'm mistaken on this point (which would not surprise me!), it would be worth adding explanation of why this isn't the case.

In practical terms I think it would be worth adding an analysis of how sensitive your results are to epsilon, and maybe also to N replicates.

Fig 4 touches on this, but since it just compares an extremely small epsilon value to a reasonable one, seems to just show that epsilon is important, without much helping to understand the best value.

I also didn't find a discussion of why 10 replicates was chosen.

I don't know that every analysis in the paper needs to be redone for multiple N, but there should at least be a discussion of the limits in N of the method's applicability.

It's geared toward "small" N, but surely there's also a lower limit, and this seems important to know from a practical standpoint.

Although they're focused on purposefully splitting up single data samples, it might be worth contextualizing with relation to these papers:

- Minsker et al. (2014, "Robust and Scalable Bayes via a Median of Subset Posteriors"): uses geometric median rather than product, but still with the goal of robustness to outliers https://arxiv.org/abs/1403.2660

- Consensus Monte Carlo approaches, such as: Scott et al. (2016, "Bayes and Big Data: The Consensus Monte Carlo Algorithm")

formatting:

Please always include line numbers in drafts.

Something seems to be wrong with the equation typesetting -- characters are often slightly misaligned.

- e.g. in the Fig 4 caption where a minus sign overlaps an equals sign

- but most or all equations are difficult to read because of the character placement

Equation numbers shouldn't have "eq.", and should be further right. They currently appear to just be part of the equation.

Fig 2

- letters indicating panels (a/b/c/d/e) need to be at least bold, if not larger; they're too inconspicuous

panels a, b, c:

- suggest to also put tick labels in a and b

- what do red/blue/green indicate? (I don't think it's the same as 2d? But even if it is, 2d doesn't have green)

- it's too hard to distinguish the colors/points -- for instance the green triangles are very hard to see, partly since they're so small that they're hidden by other points

- maybe just larger points and more transparency, but may also need to change colors. Suggest a default contrasting color palette such as 'viridis'

Fig 2d seems to over-cover, which is better than under-covering, but still not optimal

- is this related to epsilon choice?

- maybe epsilon choice should explicitly target correct coverage?

- please comment on and explain this

This sentence confuses me, I think there's some misplaced words:

"we perturb θ using for 7 replicates with moderate Gaussian noise and for 3 replicates with larger perturbations (B, Hierarchical)"

If the colors do mean the same thing in all panels, the legend needs to include all colors (green?) and be outside a panel/in first panel

- if so, need to use the exact same colors in all panels (the red/blue in a-c are different to those in the other panels)

- if not, use more different colors for different panels

Fig 3

CNV should be defined in caption

I think I'm supposed to be just visually comparing the spread of four different groups of lines here?

I can kind of tell that yellow and red are bundled more tightly than grey, but:

- the plots are too busy -- I can't see much because so many lines are on top of each other

- the hardest to see is empirical (grey)

- Suggest at least changing the empirical color to be easier to see, and/or adding transparency to all of them

- It may be better to add a lightly shaded band for each method (leave empirical as single lines), rather than plotting all curves for every method

- I don't think just looking at visual scatter is sufficient to make a claim as strong as "more closely captures the observed between-replicate variability".

- To justify this I think you'd need to quantify the variance of each method here.

Fig 4

In its current form, this figure seems more suited to earlier in the text, perhaps the introduction, since it's just establishing that espilon helps minimize the effects of outliers (i.e. providing some intuition for the equations).

I think it would be better, however, to have a comprehensive test of the effects of different epsilon choices.

Fig 5

Three extra distributions (nu, N_t, and mu) are bimodal in the collective posterior, but not in either the individual posteriors or the purple method. Is this worrisome? Is it expected?

Some of the extra peaks really don't look like they correspond to any feature in the replicate-specific data.

Why is it necessarily good that your method gives narrower posteriors?

The text quotes a formula for how much narrower, but this should be compared to the widths in the Figure, since I would guess based on the over-coverage above that they might be too narrow.

p20

This seems like the first you're mentioning this alternative concatenation approach -- how does it relate to all the other methods you mention in the introduction?

reproducibility

While I very much appreciate the authors' effort to get everything up on github, it would really benefit from a little more work to make it easier to install.

I got the code to run, but it required a lot of trial and error.

The readme says sbibm isn't required, but simulators.py imports sbibim at top level, so importing any simulator crashes without it, so I had to install it.

Following the version specifications in the readme doesn't seem to work

- sbibm requires sb < 0.22.0, but the pretrained posterior pkl files were saved with sb >= 0.23, so the pickle load calls crash

- the workaround seems to be to force-install sbi==0.23.1 after sbibm, breaking sbibm's version constraint

- the readme's install order does work (install sbibm first, then override with sbi==0.23.1), but pip warns about the incompatibility, so it would be nice to mention this

It would be great to have a requirements.txt, setup.py, or pyproject.toml, so we don't have to guess on dependencies, ideally incorporating into a pypi package.

Reviewer #2: Firstly, I apologize for the delay in returning my review. Let me say openly that I do not use LLMs, at all, in peer review. This means that it takes me much longer than many others, and there are likely elements that I may miss. I am enough of a purist that I believe that peer review should be done by humans, now at least (until the scientific community converges on a formal policy).

I enjoyed this manuscript overall and believe that an iteration of it should be fit for publication in the near future. That is, I feel like the question that it addresses is relevant; I feel like it handles most of the technical details at least reasonably well; I enjoyed the multiple use cases. But there are several issues that need to be addressed before it is suitable for publication, in my view. I’ll divide my comments into “Major” and “Minor” comments.

MAJOR COMMENTS

This manuscript addresses an important and practical problem in simulation-based inference: how to combine information from multiple empirical replicates when likelihoods are intractable and replicate variability exceeds what the generative model predicts. Put bluntly, this is one of the most important problems in all of empirical biology and is related to the highly popularized “replication crisis.” While this study may not solve this issue, it does offer some very good ideas. The paper proposes a simple and general approach that aggregates replicate-level posterior distributions into a single collective posterior using a product-of-experts formulation, with an additional mechanism designed to reduce sensitivity to outliers.

I enjoyed this paper and think the core idea is genuinely useful. Aggregating replicate-level posteriors via a product-of-experts is simple, broadly applicable, and the biological use cases make a compelling story. I'd like to see it published, but there are a few things that need addressing first.

On the methodology. The derivation of the collective posterior rests on assumptions (conditional independence of replicates, a shared prior) that are never explicitly stated. Please spell these out. Similarly, the justification for temperature scaling leans on a variance argument that only holds cleanly for Gaussians. A bit more support here, even empirical, would help.

On the benchmarking. For a methods paper, the comparison feels thin. NPE+PIE is the primary competitor, and NPSE is dismissed fairly quickly. It would strengthen the paper to engage more seriously with the broader landscape of competing approaches. Additionally, on empirical datasets, performance is largely judged through visual posterior predictive checks. Quantitative metrics would make the claims more convincing.

On the swamp sparrow reanalysis. The authors reconstruct population-specific posteriors by selecting the 100 nearest simulations from Lachlan et al.'s published output and fitting a KDE. This is a fairly rough approximation and its validity is never established. Some justification that this produces meaningful posteriors would be reassuring.

On computational efficiency. Using the prior as the SIR proposal is simple but can become very inefficient in higher dimensions. Some diagnostics like effective sample sizes would help readers assess whether this is actually practical at scale.

Overall the contribution is solid and the revisions are tractable. I look forward to seeing a revised version.

MINOR COMMENTS

There are a few rather tiny typographical and notational issues that should be corrected. For example, “stochoasticity” should read “stochasticity,” and “premutation-invariant embeddings” appears to be a typographical error for “permutation-invariant embeddings.”

Some notation could be introduced more clearly. In particular, ε is described as a minimum posterior density but the distinction between density values and probabilities is not emphasized, which may cause confusion when interpreting numerical values.

The description of the Wright–Fisher simulator would benefit from slightly clearer notation for the mutation matrix and the indexing of genotype states.

Finally, some figure descriptions could be streamlined to help readers connect the experimental design to the results shown. For example, the panels illustrating clean, hierarchical, and noisy synthetic replicate regimes would be easier to interpret if the parameter perturbations defining each regime were briefly summarized in the figure discussion.

Reviewer #3: Summary:

This paper studies inference from multiple conditionally independent replicates by aggregating replicate-specific posteriors into a collective posterior over shared parameters. The main issue is that NPE can behave less reliably in low-density regions, and naively multiplying per-replicate posteriors can let a single inconsistent replicate (perhaps due to a single outlier) dominate the result. The paper addresses this with a simple epsilon floor that limits the influence of any one replicate. Across synthetic SBI benchmarks and several empirical examples, the method is generally competitive with or better than NPE+PIE while requiring substantially less training time.

I found the paper interesting and potentially useful. The core idea is simple, seems easy to implement, and appealing from a practical SBI perspective. The manuscript also has good breadth of experiments and considers synthetic sbi examples as well as some interesting applications, and the reported training-cost advantage over NPE+PIE does seem meaningful.

Comments (note: fairly minor and mainly at author discretion):

1. Positioning of the contribution and relation to prior work.

As written, the abstract and introduction perhaps sometimes suggest that the posterior aggregation identity itself is novel. I might suggest sharpening the contribution statement, as the more novel contribution does seem to more specifically be the epsilon-floor robustification in this SBI with replications setting. It would also help to cite and contrast this approach with the paper “Gravitational wave populations and cosmology with neural posterior estimation” (Leyde et al., 2024) - an SBI approach them seems to also be considering the “Eq 1.” scenario, and (optionally) briefly situate the method relative to alternative approaches that could have been done, such as weighted or tempered PoE variants (in contrast to the epsilon flooring approach).

2. Sampling diagnostics for the SIR step.

The main results rely on prior-proposal SIR with temperature scaling and Gaussian jitter. Since the practical efficiency of this step depends on how concentrated the importance weights become, I think the paper could report a simple sampling diagnostic, such as the ESS of the resampling weights. In particular for the 10-dimensional GLU benchmark, just to check weight degeneracy isn’t an issue here.

3. Framing as robustness to misspecification.

The motivating failure mode here of replicates differing more than the simulator predicts because of batch effects, biological heterogeneity, or simulation-to-reality gap, as noted in the paper, could be viewed as a model misspecification problem, I think this could be made a bit more explicit and could connect with related work in the SBI literature. Perhaps could optionally add a couple sentences/clarifications, situation this epsilon-floor approach in that context might help readers evaluate using this approach vs other robust SBI approaches (e.g., potentially there is a connection here with generalised Bayesian inference).

Spelling and grammar

The manuscript would benefit from a careful proofread. Some errors include “and and” (p. 10), “stochoasticity” (p. 12), “sllable” (p. 14), “tighly” (p. 17), “dimensionaility” (p. 20), “premutation-invariant” (p. 21), and “Wrigh-Fisher” in the Figure 2 caption, as well as “dranatically” and “underpeforms” in the supplementary Figure S16 caption.

**********

**Have the authors made all data and (if applicable) computational code underlying the findings in their manuscript fully available?**

The PLOS Data policy requires authors to make all data and code underlying the findings described in their manuscript fully available without restriction, with rare exception (please refer to the Data Availability Statement in the manuscript PDF file). The data and code should be provided as part of the manuscript or its supporting information, or deposited to a public repository. For example, in addition to summary statistics, the data points behind means, medians and variance measures should be available. If there are restrictions on publicly sharing data or code —e.g. participant privacy or use of data from a third party—those must be specified.

Reviewer #1: Yes

Reviewer #2: None

Reviewer #3: Yes

**********

PLOS authors have the option to publish the peer review history of their article (what does this mean?). If published, this will include your full peer review and any attached files.

If you choose “no”, your identity will remain anonymous but your review may still be made public.

**Do you want your identity to be public for this peer review?**  For information about this choice, including consent withdrawal, please see our Privacy Policy.

Reviewer #1: No

Reviewer #2: No

Reviewer #3: No

[NOTE: If reviewer comments were submitted as an attachment file, they will be attached to this email and accessible via the submission site. Please log into your account, locate the manuscript record, and check for the action link "View Attachments". If this link does not appear, there are no attachment files.]

**Figure resubmission:**

While revising your submission, we strongly recommend that you use PLOS’s NAAS tool (https://ngplosjournals.pagemajik.ai/artanalysis) to test your figure files. NAAS can convert your figure files to the TIFF file type and meet basic requirements (such as print size, resolution), or provide you with a report on issues that do not meet our requirements and that NAAS cannot fix.

After uploading your figures to PLOS’s NAAS tool - https://ngplosjournals.pagemajik.ai/artanalysis, NAAS will process the files provided and display the results in the "Uploaded Files" section of the page as the processing is complete. If the uploaded figures meet our requirements (or NAAS is able to fix the files to meet our requirements), the figure will be marked as "fixed" above. If NAAS is unable to fix the files, a red "failed" label will appear above. When NAAS has confirmed that the figure files meet our requirements, please download the file via the download option, and include these NAAS processed figure files when submitting your revised manuscript.

**Reproducibility:**

To enhance the reproducibility of your results, we recommend that authors of applicable studies deposit laboratory protocols in protocols.io, where a protocol can be assigned its own identifier (DOI) such that it can be cited independently in the future. Additionally, PLOS ONE offers an option to publish peer-reviewed clinical study protocols. Read more information on sharing protocols at https://plos.org/protocols?utm_medium=editorial-email&utm_source=authorletters&utm_campaign=protocols

# Author comment: Author response to Decision Letter 1

**DOI:** 10.1371/journal.pcbi.1014534.r002

3 Jun 2026

Attachment Submitted filename: response letter - collective posterior.pdf

# Peer review: Decision Letter 1

**DOI:** 10.1371/journal.pcbi.1014534.r003

**Authors:** Tobias Bollenbach, Raymond Louie

5 Jul 2026

Dear Dr. Ram,

We are pleased to inform you that your manuscript 'Collective Posterior Inference from Highly Variable Empirical Replicates' has been provisionally accepted for publication in PLOS Computational Biology.

Before your manuscript can be formally accepted you will need to complete some formatting changes, which you will receive in a follow up email. A member of our team will be in touch with a set of requests.

Please note that your manuscript will not be scheduled for publication until you have made the required changes, so a swift response is appreciated.

IMPORTANT: The editorial review process is now complete. PLOS will only permit corrections to spelling, formatting or significant scientific errors from this point onwards. Requests for major changes, or any which affect the scientific understanding of your work, will cause delays to the publication date of your manuscript.

Should you, your institution's press office or the journal office choose to press release your paper, you will automatically be opted out of early publication. We ask that you notify us now if you or your institution is planning to press release the article. All press must be co-ordinated with PLOS.

Thank you again for supporting Open Access publishing; we are looking forward to publishing your work in PLOS Computational Biology.

Best regards,

Raymond Louie

Academic Editor

PLOS Computational Biology

Tobias Bollenbach

Section Editor

PLOS Computational Biology

***********************************************************

Reviewer's Responses to Questions

**Comments to the Authors:**

**Please note here if the review is uploaded as an attachment.**

Reviewer #1: Thanks for the detailed comments.

Reviewer #2: My comments have been addressed

Reviewer #3: The authors have addressed my three previous comments quite satisfactorily. I have no further requests and recommend acceptance.

**********

**Have the authors made all data and (if applicable) computational code underlying the findings in their manuscript fully available?**

The PLOS Data policy requires authors to make all data and code underlying the findings described in their manuscript fully available without restriction, with rare exception (please refer to the Data Availability Statement in the manuscript PDF file). The data and code should be provided as part of the manuscript or its supporting information, or deposited to a public repository. For example, in addition to summary statistics, the data points behind means, medians and variance measures should be available. If there are restrictions on publicly sharing data or code —e.g. participant privacy or use of data from a third party—those must be specified.

Reviewer #1: None

Reviewer #2: None

Reviewer #3: None

**********

PLOS authors have the option to publish the peer review history of their article (what does this mean?). If published, this will include your full peer review and any attached files.

If you choose “no”, your identity will remain anonymous but your review may still be made public.

**Do you want your identity to be public for this peer review?**  For information about this choice, including consent withdrawal, please see our Privacy Policy.

Reviewer #1: No

Reviewer #2: No

Reviewer #3: No

# Editor report: Acceptance letter

**DOI:** 10.1371/journal.pcbi.1014534.r004

**Authors:** Tobias Bollenbach, Raymond Louie

PCOMPBIOL-D-26-00248R1

Collective Posterior Inference from Highly Variable Empirical Replicates

Dear Dr Ram,

I am pleased to inform you that your manuscript has been formally accepted for publication in PLOS Computational Biology. Your manuscript is now with our production department and you will be notified of the publication date in due course.

The corresponding author will soon be receiving a typeset proof for review, to ensure errors have not been introduced during production. Please review the PDF proof of your manuscript carefully, as this is the last chance to correct any errors. Please note that major changes, or those which affect the scientific understanding of the work, will likely cause delays to the publication date of your manuscript.

Soon after your final files are uploaded, unless you have opted out, the early version of your manuscript will be published online. The date of the early version will be your article's publication date. The final article will be published to the same URL, and all versions of the paper will be accessible to readers.

For Research, Software, and Methods articles, you will receive an invoice from PLOS for your publication fee after your manuscript has reached the completed accept phase. If you receive an email requesting payment before acceptance or for any other service, this may be a phishing scheme. Learn how to identify phishing emails and protect your accounts at https://explore.plos.org/phishing.

Thank you again for supporting PLOS Computational Biology and open-access publishing. We are looking forward to publishing your work!

With kind regards,

Sharmila Kamatchi

PLOS Computational Biology | Carlyle House, Carlyle Road, Cambridge CB4 3DN | United Kingdom ploscompbiol@plos.org | Phone +44 (0) 1223-442824 | ploscompbiol.org | @PLOSCompBiol
